# Whole exome analysis of patients in Japan with hearing loss reveals high heterogeneity among responsible and novel candidate genes

**DOI:** 10.1186/s13023-022-02262-4

**Published:** 2022-03-05

**Authors:** Hideki Mutai, Yukihide Momozawa, Yoichiro Kamatani, Atsuko Nakano, Hirokazu Sakamoto, Tetsuya Takiguchi, Kiyomitsu Nara, Michiaki Kubo, Tatsuo Matsunaga

**Affiliations:** 1 Division of Hearing and Balance Research, National Institute of Sensory Organs, National Hospital Organization Tokyo Medical Center, 2-5-1 Higashigaoka, Meguro-ku, Tokyo 152-8902 Japan; 2 Laboratory for Genotyping Development, RIKEN Center for Integrative Medical Sciences, 1-7-22 Suehiro-cho, Tsurumi-ku, Yokohama, Kanagawa 230-0045 Japan; 3 Laboratory for Statistical Analysis, RIKEN Center for Integrative Medical Sciences, 1-7-22 Suehiro-cho, Tsurumi-ku, Yokohama, Kanagawa 230-0045 Japan; 4 Department of Otorhinolaryngology, Chiba Children’s Hospital, 579-1 Hetacho, Midori-ku, Chiba-shi, Chiba 266-0007 Japan; 5 Department of Otorhinolaryngology, Hyogo Prefectural Kobe Children’s Hospital, 1-6-7 Minatojima Minami-cho, Chuo-ku, Kobe-shi, Hyogo 650-0047 Japan; 6 Present Address: Department of Otolaryngology, Graduate School of Medicine, Osaka City University, 1-4-3, Asahimachi, Abeno-ku, Osaka-shi, Osaka 558-8585 Japan; 7 Department of Otolaryngology, National Hospital Organization Kanazawa Medical Center, 1-1 Shimoishibiki-machi, Kanazawa-shi, Ishikawa 920-8650 Japan; 8 RIKEN Center for Integrative Medical Sciences, 1-7-22 Suehiro-cho, Tsurumi-ku, Yokohama-shi, Kanagawa 230-0045 Japan; 9 Medical Genetics Center, National Hospital Organization Tokyo Medical Center, 2-5-1 Higashigaoka, Meguro-ku, Tokyo 152-8902 Japan

**Keywords:** Whole exome sequencing analysis, Hearing loss, Deafness genes

## Abstract

**Background:**

Heterogeneous genetic loci contribute to hereditary hearing loss; more than 100 deafness genes have been identified, and the number is increasing. To detect pathogenic variants in multiple deafness genes, in addition to novel candidate genes associated with hearing loss, whole exome sequencing (WES), followed by analysis prioritizing genes categorized in four tiers, were applied.

**Results:**

Trios from families with non-syndromic or syndromic hearing loss (n = 72) were subjected to WES. After segregation analysis and interpretation according to American College of Medical Genetics and Genomics guidelines, candidate pathogenic variants in 11 previously reported deafness genes (*STRC*, *MYO15A*, *CDH23*, *PDZD7*, *PTPN11*, *SOX10*, *EYA1*, *MYO6*, *OTOF*, *OTOG*, and *ZNF335*) were identified in 21 families. Discrepancy between pedigree inheritance and genetic inheritance was present in one family. In addition, eight genes (*SLC12A2*, *BAIAP2L2*, *HKDC1*, *SVEP1*, *CACNG1*, *GTPBP4*, *PCNX2*, and *TBC1D8*) were screened as single candidate genes in 10 families.

**Conclusions:**

Our findings demonstrate that four-tier assessment of WES data is efficient and can detect novel candidate genes associated with hearing loss, in addition to pathogenic variants of known deafness genes.

**Supplementary Information:**

The online version contains supplementary material available at 10.1186/s13023-022-02262-4.

## Background

Approximately 1 in every 500 newborns exhibits a degree of hearing loss, and more than half of cases are associated with genetic mutations [1]. Genes responsible for hereditary hearing loss are highly heterogeneous. Recent advances in clinical genome sequencing, focusing on known deafness gene panels, have been used to efficiently detect pathogenic variants, inform appropriate clinical intervention (such as cochlear implants), and estimate prognosis, in terms of symptoms [2–4]. To date, more than 100 genes have been reported as associated with non-syndromic hearing loss [5]. Further, according to Online Mendelian Inheritance in Man (OMIM), hundreds of genes are associated with syndromic hearing loss. Targeted resequencing of deafness genes is cost-effective and, therefore, beneficial for diagnostic purposes [3]; however, it is not suitable for detection of very rare or novel deafness genes.

Whole exome sequencing (WES), involves sequencing of coding exons comprising approximately 2% of the whole human genome, which are estimated to contain approximately 85% of pathogenic variants associated with monogenic disease [6]. For efficient identification of pathogenic variants in patient samples by analysis of WES data, detected variants are often categorized in several groups, where those in genes previously associated with the clinical features of interest are the first priority for analysis [7, 8]. Sets of prioritized genes can be modified during analysis to increase the number of targeted genes, without resequencing the same samples. For comprehensive investigation of the genetic heterogeneity of diseases with a wide range of causative genes, such as hearing loss, and to identify novel candidate genes, WES analysis overcomes the limitations of targeted analysis and is considerably more cost-effective than whole genome sequencing (WGS) analysis.

In this study, we sought to explore the wide spectrum of genetic heterogeneity associated with hearing loss in Japan, and to discover novel candidate genes associated with hearing loss, using trio analysis of probands and their parents, and four originally developed gene groups ranked by priority (tiers), as a new strategy to filter candidate variants. Using this strategy, we successfully detected candidate pathogenic variants in 11 previously reported deafness genes in 21 families, as well as eight single candidate deafness genes in 10 families.

## Methods

### Editorial policies and ethical considerations

The Ethics Review Committees of the National Hospital Organization Tokyo Medical Center (approval number: R1-0703009) and all collaborating institutes approved the study procedures. All procedures were conducted after written informed consent had been obtained from each subject or their parents.

### Subjects

All subjects were patients visiting the National Hospital Organization Tokyo Medical Center or collaborating hospitals. Medical histories were obtained, and clinical information, such as the results of physical, audiological, and blood tests, were collected from subjects and family members, when available. Hearing loss severity was determined according to the recommendations of the Genetic Deafness study group, using audiological tests, including pure-tone audiometry, auditory steady-state response, conditioned orientation reflex audiometry, or play audiometry, depending on the age of the patient and availability [9]. Subjects with hearing loss related to environmental factors, such as meningitis, premature birth, and rubella, were excluded.

### Genetic analysis

Genomic DNA was obtained from blood samples collected from probands and their family members, mostly parents. Probands with known high prevalence deafness gene variants, and those with specific clinical features suggesting subsets of deafness genes, were filtered using the following methods. All probands were screened for *GJB2* or mitochondrial m.1555A>G and m.3243A>G variants, which are frequently detected in Japanese patients with hereditary hearing loss, as described previously [10]. Probands were also screened for *SLC26A4* variants when enlarged vestibular aqueduct was detected by computed tomography (CT), or when they were not examined by CT. Probands with auditory neuropathy, which manifests as normal otoacoustic emission and loss of auditory brainstem responses, were subjected to Sanger sequencing analysis of *OTOF* [11]. To rule out congenital cytomegalovirus infection, PCR examination for cytomegalovirus in the preserved umbilical cords of probands was conducted, when samples were available.

WES protocols have been reported previously [12]. In brief, genomic DNA extracted from blood was subjected to whole exome region capture using a Nextera Rapid Capture Exome kit (Illumina) [13] and to massively parallel sequencing using the HiSeq2500 platform (Illumina). Sequence reads were mapped onto the human reference genome (GRCh37) with a decoy sequence (hs37d5), using BWE-mem (v.0.7.5a), and variants were called using the Picard (v.1.106) and Genome Analysis Toolkit 3.4.46 (GATK) [14]. Individual variants were joint-called, together with in-house data (WES, *n* = 498 and WGS, *n* = 1037) [15] using GenotypeGVCFs. Variants were then annotated using Annovar [16]. Variants in repeat elements, low complexity regions, or considered to result from strand bias, were omitted from further analyses. Average mapping rate, read depth, and numbers of SNVs and indels, are presented in Additional file 1.

A schematic flowchart of the WES analysis conducted in this study is shown in Additional file 2. To identify candidate pathogenic changes, variants predicted to alter the encoded protein were first filtered according to minor allele frequency (MAF), as previously descried [12]. In brief, a threshold MAF of < 0.001 was applied for AD inheritance mode analysis of global public databases (Database of Single Nucleotide Polymorphisms (dbSNP) [17], East Asian population of 1000 Genomes [18], NHLBI Exome Variant Server (ESP6500), Exome Aggregation Consortium (ExAC) [19], Genome Aggregation Database (gnomAD) [20], Human Genetic Variation Database (HGVD) ver1.42 based on 1208 healthy Japanese subjects [21], and an in-house database including 1037 healthy Japanese subjects [15]; and a MAF threshold of < 0.003 applied for sporadic cases and AR inheritance mode analysis of global databases, except that a threshold of < 0.005 was used for the HGVD and in-house databases, as previously described [12]. Variants were further excluded out from candidates if all the in silico analyses (LRT, LR, Mutation Assessor, Mutation Taster, Polyphen 2-HDIV, Polyphen 2-HVAR, RadialSVM, SIFT) predicted no, benign, or tolerated effect of the variant. The effect of the splice site variants was predicted by MaxEntScan [22] and Human Splice Finder 3.0 [23] with default threshold values.

Remaining variants were prioritized in four categories before analysis of co-segregation with the disease: (1) Tier 1 genes were reported as associated with non-syndromic, syndromic hearing loss, and diseases including hearing loss as a non-characteristic symptom registered in OMIM (n = 293, gene list in [12]); (2) Tier 2 genes were associated with hearing loss in animal models by the Mouse Genome Informatics [24] or International Mouse Phenotyping Consortium [25], and not included in Tier 1 (n = 328, gene list in [12]); and (3) Tier 3 genes were expressed at > twofold higher levels in *M. fascicularis* cochlea than in other tissues [26] and were not included in Tier 1 or Tier 2 (n = 305; Additional file 3). Genes with high expression levels in *M. fascicularis* cochlea are enriched for deafness genes and may therefore contain novel candidates [26]. In total, 926 genes were categorized in Tiers 1–3. Genes not included in Tiers 1–3 were categorized as Tier 4.

Among selected variants co-segregating with hearing loss, those in Tier 1 genes were searched in the OMIM, Human Genome Mutation Database (HGMD) (last accessed March 12, 2019) and ClinVar (last accessed March 12, 2019) to determine the consistency of the clinical features of the individuals in this study, according to PP4 criterion in the ACMG guidelines [27]. Genes associated with syndromic hearing loss were excluded if they met the following criteria: (1) the variants had not been reported as pathogenic or likely pathogenic, and (2) the proband did not exhibit the characteristic symptoms of multiple organ disease caused by that gene.

Remaining candidate variants were subjected to PCR and Sanger sequencing. Primer sets used in this study are shown in Additional file 5. Representative electropherograms of variants detected in each proband are shown in Additional file 6 and Additional file 10.

### Assessment of large deletion allele of *STRC*

A suspected homozygous deletion of *STRC*, mapping to chromosome 15q15.3 [28, 29], and detected in patients using IGV [30], was validated by MLPA (kit P461, MRC-Holland, Amsterdam, Netherlands), according to the manufacturer’s protocols. Copy numbers of exon 19 and the 5′ flanking region of *STRC* were also examined by duplicated quantitative PCR (qPCR) in subjects and their family members. Primers for copy number quantification of the exon 10 region of *MYO7A* (NM_000260.3) were used as a reference. Primer sets used in this study are presented in Additional file 5.

## Results

### Overview of subjects

Seventy-two families including 215 individuals (71 families with the proband and parents, and one with the proband and mother) were recruited for this study (Table 1). Most of the participants were Japanese, while a father with normal hearing in one family was Korean. Within the families, the majority of probands appeared to be sporadic cases (52 families, 72%). In addition, 9 (13%) and 10 (14%) families were presumed to have autosomal-dominant (AD) and autosomal-recessive (AR) inheritance modes, respectively, based on the symptoms of family members. The inheritance mode was not determined in one family, since the proband and both parents had hearing loss. The majority of probands had non-syndromic hearing loss (58 families, 81%).

**Table 1:** Number of families participated in this study

Number of family	Sporadic	Autosomal dominant	Autosomal recessive	Undetermined
Nonsyndromic	41 (20)	8 (4^†^)	8 (2)	1^‡^ (0)
Syndromic	11 (3)	1 (1)	2 (1)	0 (0)

Number in partenthesis indicate the families whose candidate responsible gene was narrowed down to one

^†^Two responsible genes in 2 cases were identified in a family (family 1633)

^‡^Parents and proband all show hearing loss

### Detection of candidate pathogenic variants in previously known deafness genes

In WES analysis, a mean read depth of approximately 144 with > 99.9% average mapping rate, was obtained (Additional file 1). Approximately 0.4% of the targeted regions (848 out of 212,158 regions) showed insufficient read depth (< 20) consistently. Among them, 5 regions were included in Tier 1 genes (Additional file 4). In Tier 1 genes, approximately 0.67% of targeted bases (4,644 out of 697,091 bases) showed insufficient read depth consistently.

**Fig. 1:**
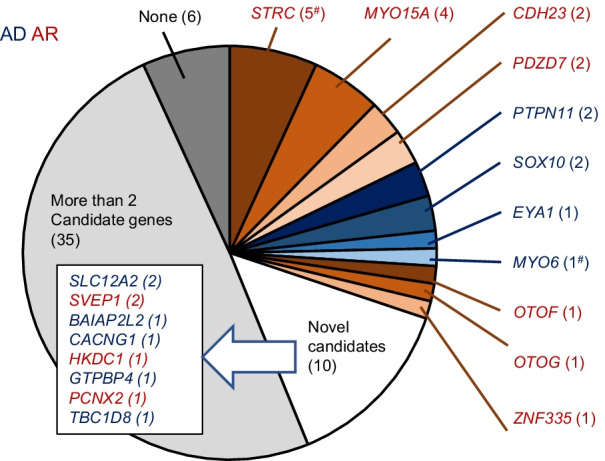
Frequencies of identified candidate genes associated with hearing loss in this study. Numbers in parenthesis indicate families carrying a candidate gene. Genes in red indicate those consistent with an autosomal recessive (AR) inheritance mode, and those in blue indicate genes consistent with an autosomal dominant (AD) inheritance mode. Sharp symbols (#) indicate that two deafness genes were found in different members of one family

By trio analysis, 11 previously reported deafness genes were considered to be responsible for hearing loss in 21 families (Fig. 1). As described in “Methods” section, probands were prescreened for *GJB2* variants, including the m.1555 A>G and m.3243 A>G variants, as well as *SLC26A4* and *OTOF* variants, depending on their clinical features. All detected genes, genotypes, diseases, and clinical features of probands are summarized in Table 2. Additional bioinformatic data for each variant, including allele frequencies in population databases, in silico analyses, and conservation among vertebrate species, are shown in Table 3. Partial Sanger sequencing electropherograms validating each variant are presented in Additional file 6. All the variants reported in this study fulfilled at least one criterion (PM2_Supporting, absent or extremely low frequency in population databases), according to the American College of Medical Genetics and Genomics (ACMG) guidelines [27] and the modification of PM2 by ClinGen Sequence Variant Interpretation Working Group (https://www.clinicalgenome.org/site/assets/files/5182/pm2_-_svi_recommendation_-_approved_sept2020.pdf).

**Table 2:** Clinical features of probands and genotypes of known deafness genes

Family ID	Gene symbol	Disease	Reference mRNA	Genotype-nucleotide change	Predicted inheritance mode of family	Sex	Hearing levels (left/right)	Audiogram (L/R)
1470	*MYO15A*	DFNB3	NM_016239.3	c.[419delA];[1185dupC]	Sporadic	F	Severe/severe^†^	Flat/gently sloping^†^
1540	*MYO15A*	DFNB3	NM_016239.3	c.[419delA];[9938A>C]	Sporadic	F	Severe/profound	Gently sloping/gently sloping
1479	*MYO15A*	DFNB3	NM_016239.3	c.[8450G>C];[9690+1G>A]	Sporadic	M	Moderate	Flat
1688	*MYO15A*	DFNB3	NM_016239.3	c.[1185dupC];[8969delG]	Sporadic	F	Severe/severe	Sloping/sloping
1644	*CDH23*	DFNB12/USH1D	NM_022124.5	c.[719C>T];[805C>T]	Sporadic	M	Profound/profound^†^	Steep sloping/sloping^†^
1528	*CDH23*	DFNB12/USH1D	NM_022124.5	c.[719C>T];[7802T>C]	AR	M	Severe/severe	Steep sloping/steep sloping
1397	*PDZD7*	DFNB57	NM_001195263.1	c.[490C>T];[503G>C]	AR	F	Moderate/moderate	Sloping/sloping
1597	*PDZD7*	DFNB57	NM_001195263.1	c.[490C>T];[494G>C]	AR	F	Moderate/moderate	Sloping/gently sloping
1648	*OTOF*	DFNB9	NM_001287489.1	c.[5816G>A];[5816G>A]	Sporadic	F	Severe	Flat
739	*OTOG*	DFNB18B	NM_001277269.1	c.[2116+2_2116+12del11];[5425C>T]	Sporadic	M	Moderate/moderate^†^	Flat/flat^†^
1631	*PTPN11*	NS1	NM_002834.3	c.[836A>G];[=]	Sporadic	M	Severe/severe^†^	Flat/flat^†^
1543	*PTPN11*	NS1	NM_002834.3	c.[1529A>G];[=]	Sporadic	F	Severe/profound^†^	Flat/steep sloping^†^
1583	*SOX10*	WS2E/4C/PCWH	NM_006941.3	c.[570C>A];[=]	Sporadic	F	Severe/severe	Gently sloping/gently sloping
1651	*SOX10*	WS2E/4C/PCWH	NM_006941.3	c.[1122delC];[=]	Sporadic	M	Severe/profound	Flat/gently sloping
1636	*EYA1*	BOS1/BOR	NM_000503.5	c.[1082G>A];[=]	AD	M	Profound/profound^†^	Flat/flat^†^
1456	*ZNF335*	PMCH10	NM_022095.3	c.[1399T>C];[1505A>G]	Sporadic	F	Severe	Flat
1410	*STRC*	DFNB16	NM_153700.2	c.[(?_ -1)_(*1_?)del];[(?_-1)_(*1_?)del]	AR	M	Profound/severe	Flat/flat
1564	*STRC*	DFNB16	NM_153700.2	c.[(?_-1)_(*1_?)del];[(?_-1)_(*1_?)del]	Sporadic	F	Mild/moderate	Sloping/sloping
1700	*STRC*	DFNB16	NM_153700.2	c.[(?_-1)_(*1_?)del];[(?_-1)_(*1_?)del]	Sporadic	M	Moderate/moderate	Flat/flat
1436	*STRC*	DFNB16	NM_153700.2	c.[(?_-1)_(*1_?)del];[(?_-1)_(*1_?)del]	Sporadic	F	Moderate	Flat to gently sloping
1633 (I-2)	*STRC*	DFNB16	NM_153700.2	c.[(?_-1)_(*1_?)del];[(?_-1)_(*1_?)del]	AD^‡^	F	Moderate/moderate	Gently sloping/gently sloping
1633 (II-1)	*MYO6*	DFNA22	NM_004999.3	c.[1325G>A];[=]	AD^‡^	M	Moderate/moderate	2k dip/U-shape

Family ID	Onset age of hearing loss (y)	Fluctuation	Progression	Tinnitus	Vestibular defect	Inner ear morphology (CT or MRI)	Additional phenotypes
1470	0	No	No	Unknown	No	Normal	
1540	0	No	No	Unknown	No	Normal	
1479	0	No	Yes	Unknown	Unknown	Normal	
1688	< 4	No	Yes	Unknown	Unknown	Normal	
1644	0	No	No	No	No	Normal	Motor develomental delay
1528	0	No	Unknown	No	No	Normal	
1397	0	No	No	Unknown	Unknown	Normal	The sibling of proband shows moderate to severe hearing loss
1597	0	No	No	Unknown	No	Normal	The sibling of proband shows congenital, moderate hearing loss with high frequency–sloping type of audiograms
1648	0	No	No	No	No	Normal	Otoacoustic emission not tested
739	0	No	No	Unknown	No	Normal	
1631	0	No	No	No	No	Normal	Short statue with subtle ocular hypertelorism, café au-lait pigmentation. Intellectual development not evaluated
1543	0	Unknown	No	Unknown	Unknown	Normal	
1583	0	No	No	Unknown	Yes	Enlarged inner ear canals, Insufficient cochlear turn, hypoplasia of bilateral semicircular canals	Mixed hearing loss and vertigo
1651	0	No	Yes	Unknown	Yes	Normal	Ptosis on right eye, brown eyes, motor development delay. Normal digestive tract
1636	0	No	Yes	No	No	Not tested	Amblyopia with refractive errors, occasional cloudy urine. The father showed mild, low-frequency bilateral hearing loss. No branchial abnormalities
1456	0	Unknown	Unknown	Unknown	Unknown	Brain hypoplasia	Epilepsy, brain hypoplasia, hypertonia, convulsion, poor weight gain, motor develomental delay
1410	9	Yes	Yes	No	No	Normal	Spiral-shaped field, progressive vision loss, hyperactivity. The sibling (III-2) exhibited progressive hearing loss with vision loss
1564	0	No	No	No	No	Normal	
1700	0	No	No	No	Yes	Normal	
1436	0	No	No	Unknown	Unknown	Not tested	Short statue
1633 (I-2)	0	Unknown	Unknown	Unknown	Unknown	Not tested	
1633 (II-1)	0	No	No	No	No	Normal	

^†^Based on ASSR

^‡^Family 1633 was initially considered to be in autosomal dominant inheritance mode; the mother and the proband was found to have distinct candidate genes responsible for hearing loss

**Table 3:** Deafness gene variants and annotation information in this study

Gene symbol	Disease	Reference mRNA	Nucleotide change	Amino acid change	Genomic position (GRCh37)	dbSNP ID	MAF (1000G)	MAF (ESP6500)	MAF (ExAC)
*MYO15A*	DFNB3	NM_016239.3	c.419del	p.(Lys140SerfsTer304)	NC_000017.10:g.18022533del	rs750130520	0	0	8.38E−06
*MYO15A*	DFNB3	NM_016239.3	c.1185dup	p.(Glu396ArgfsTer36)	NC_000017.10:g.18023299dup	rs876657707	0	5.04E−04	1.49E−04
*MYO15A*	DFNB3	NM_016239.3	c.8450G>C	p.(Arg2817Pro)	NC_000017.10:g.18058737G>C	rs761861080	0	0	8.26E−06
*MYO15A*	DFNB3	NM_016239.3	c.8969del	p.(Gly2990ValfsTer44)	NC_000017.10:g.18061838del	–	0	0	0
*MYO15A*	DFNB3	NM_016239.3	c.9690+1G>A	p.? (splice mut)	NC_000017.10:g.18066636G>A	–	0	0	0
*MYO15A*	DFNB3	NM_016239.3	c.9938A>C	p.(His3313Pro)	NC_000017.10:g.18069825A>C	–	0	0	0
*CDH23*	DFNB12/USH1D	NM_022124.5	c.719C>T	p.(Pro240Leu)	NC_000010.10:g.73330641C>T	rs121908354	0	0	0
*CDH23*	DFNB12/USH1D	NM_022124.5	c.7802T>C	p.(Val2601Ala)	NC_000010.10:g.73563107T>C	–	0	0	0
*CDH23*	DFNB12/USH1D	NM_022124.5	c.805C>T	p.(Arg269Trp)	NC_000010.10:g.73337722C>T	rs936479651	0	0	0
*PDZD7*	DFNB57	NM_001195263.1	c.490C>T	p.(Arg164Trp)	NC_000010.10:g.102783245G>A	rs200664140	0	0	4.94E−05
*PDZD7*	DFNB57	NM_001195263.1	c.494G>C	p.(Arg165Pro)	NC_000010.10:g.102783241C>G	–	0	0	0
*PDZD7*	DFNB57	NM_001195263.1	c.503G>C	p.(Arg168Pro)	NC_000010.10:g.102783232C>G	–	0	0	0
*OTOF*	DFNB9	NM_001287489.2	c.5816G>A	p.(Arg1939Gln)	NC_000002.11:g.26681086C>T	rs201326023	0	0	2.54E−05
*OTOG*	DFNB18B	NM_001277269.1	c.2116+2_2116+12del	p.? (splice mut)	NC_000011.9:g.17593753_17593763del	–	A	0	0
*OTOG*	DFNB18B	NM_001277269.1	c.5425C>T	p.(Gln1809Ter)	NC_000011.9:g.17632236C>T	–	0	0	0
*PTPN11*	NS1	NM_002834.3	c.836A>G	p.(Tyr279Cys)	NC_000012.11:g.112910827A>G	rs121918456	0	0	0
*PTPN11*	NS1	NM_002834.4	c.1529A>G	p.(Gln510Arg)	NC_000012.11:g.112926909A>G	rs121918470	0	0	8.24E−06
*SOX10*	WS 2E/4C/PCWH	NM_006941.3	c.570C>A	p.(Cys190Ter)	NC_000022.10:g.38374001G>T	–	0	0	0
*SOX10*	WS 2E/4C/PCWH	NM_006941.3	c.1122del	p.(Thr375ProfsTer127)	NC_000022.10:g.38369782del	–	0	0	0
*EYA1*	BOR, BOS	NM_000503.5	c.1082G>A	p.(Arg361Gln)	NC_000008.10:g.72156896C>T	rs145219836	0.001	0	4.12E−05
*ZNF335*	MCPH10	NM_022095.3	c.1399T>C	p.(Cys467Arg)	NC_000020.10:g.44592246A>G	–	0	0	0
*ZNF335*	MCPH10	NM_022095.3	c.1505A>G	p.(Tyr502Cys)	NC_000020.10:g.44592140T>C	rs753888773	0	0	8.24E−06
*STRC*	DFNB16	NM_153700.2	c.(?_-1)_(*1_?)del	–	–	–	–	–	–
*MYO6*	DFNA22	NM_004999.3	c.1325G>A	p.(Cys442Tyr)	NC_000006.11:g.76566915G>A	rs121912557	0	0	0

Gene symbol	MAF (gnomAD)	MAF (HGVD)	MAF (in house)	REVEL^†^	CADD^‡^	UCSC conservation^§^	Splice site (MaxEntScan)	Splice site (HSF3.1)	ACMG criteria	References (PubMED)
*MYO15A*	8.40E−06	0	0	–	–	–	–	–	PVS1, PM2_Supporting	4783301
*MYO15A*	0	0	0	–	–	–	–	–	PVS1, PM2_Supporting, PP1	3534775
*MYO15A*	4.08E−06	0	0	0.772	26.7	12/12, 41/47, 28/29	–	–	PVS1, PS1, PM2_Supporting, PM5	This study
*MYO15A*	0	0	0	–	–	–	–	–	PVS1, PM2, PM2_Supporting, PP1	This study
*MYO15A*	0	0	0	–	32	12/12, 48/48, 28/29	8.59 to 0.41	Affected (− 32.17%)	PVS1, PM2_Supporting	4444116
*MYO15A*	0	0	0	0.83	27.3	12/12, 49/49, 35/36	–	–	PM2_Supporting, PP3	This study
*CDH23*	0.00004062	0.002	0.003	0.516	29.5	11/11, 49/49, 36/36	–	–	PM2_Supporting, PP1	17850630, 4451718
*CDH23*	0	0	0	0.777	26.4	12/12, 47/47, 36/37	–	–	PM2_Supporting, PM3	This study
*CDH23*	0.000008749	0	0	0.439	27.4	12/12, 49/49, 30/30	–	–	PM2_Supporting, PM3	This study
*PDZD7*	5.28E−05	0.002	0	0.377	24.1	12/12, 49/49, 37/37	–	–	PM2_Supporting, PP1	31454969
*PDZD7*	0.000004064	0	0	0.622	29.4	12/12, 49/49, 37/37	–	–	PM2_Supporting, PM3	This study
*PDZD7*	0	0	0	0.123	25.1	12/12, 49/49,17/38	–	–	PM2_Supporting, PM3	This study
*OTOF*	3.38E−05	0.002	0	0.86	20.9	11/11, 37/48, 31/34	–	–	PM2_Supporting, PP1, PP3	1735255, 22575033
*OTOG*	0	0	0	.	.	–	10.90 to − 9.39	Affected (− 52.79%)	PVS1, PM2_Supporting	This study
*OTOG*	0	0	0	.	40	–	–	–	PVS1, PM2_Supporting	This study
*PTPN11*	0	0	0	0.973	31	12/12, 49/49, 32/32	–	–	PS1, PS2, PM2_Supporting, PP4	11992261
*PTPN11*	4.06E−06	0	0	0.959	28	12/12, 49/49, 37/37	–	–	PS1, PS2, PM1, PM2_Supporting, PM5, PP1, PP3, PP5	15948193
*SOX10*	0	0	0	0.211	28.4	–	–	–	PVS1, PS2, PM2_Supporting, PP4	This study
*SOX10*	0	0	0	.	.	–	–	–	PVS1, PS2, PM2_Supporting, PP4	This study
*EYA1*	4.08E−05	0	0	0.642	24	12/12, 48/48, 38/38	–	–	PM1, PM2_Supporting, PP1	This study
*ZNF335*	0	0	0	0.808	29	–	–	–	PM2_Supporting	27540107
*ZNF335*	8.14E−06	0	0	0.466	29.1	–	–	–	PM2_Supporting	27540107
*STRC*	–	–	–	–	–	–	–	–	PVS1	26011646, 27469136
*MYO6*	0	0	0	0.959	29.8	12/12, 50/50, 36/36	–	–	PS1, PS2, PM2_Supporting, PP3	1235492

^†^Rare exome variant ensemble learner

^‡^Combined annotation dependent depletion (v1.4)

^§^Identity of amino acid residues (nucleotide in case of splice site variant) in up to 12 primate, 50 mammalian, and 38 vertebrate species

Regarding non-syndromic deafness genes, compound heterozygous variants of *MYO15A* were identified in four sporadic cases (families 1470, 1540, 1479, and 1688); compound heterozygous variants of *CDH23* in two sporadic cases (families 1644 and 1528); and compound heterozygous variants of *PDZD7* in families 1397 and 1597; all three candidate *PDZD7* variants mapped to exon 4 in regions encoding one of the PDZ domains, which are structural anchors that tether the protein to cytoskeletal components [31]. Although *ADGRV1* and *PDZD7* have been proposed as genes responsible for Usher syndrome type IIC [32], no candidate variants of *ADGRV1* were detected among our patients. A homozygous variant of *OTOF* was identified in a sporadic case in family 1648. The pathogenicity of the c.5816G>A (p.Arg1939Gln) variant is established [11, 33]. As the proband had not been tested for otoacoustic emission, which is necessary to detect auditory neuropathy, this case was subjected to WES without prescreening for *OTOF*. Compound heterozygous variants of *OTOG* were identified in a sporadic case from family 739; the variants were predicted to disrupt splicing at the donor site (5′ splice site) of exon 11 and to be a nonsense mutation. Loss-of-function of both alleles of *OTOG* was considered to be sufficient explanation for hearing impairment.

Regarding syndromic deafness genes, two de novo variants of *PTPN11* were identified in sporadic cases in families 1631 and 1543. Both detected variants, c.836A>G (p.Tyr279Cys) and c.1529A>G (p.Gln510Arg), reside in regions encoding the catalytic sites of the non-receptor type protein-tyrosine phosphatase [34], and are established pathogenic variants causing Noonan syndrome 1 (NS1) [35, 36]. The proband in family 1631 showed syndromic symptoms (short statue with subtle ocular hypertelorism, café -au-lait pigmentation, Table 2). Although evaluation of the developmental status of the proband was limited because of the age at the time of genetic test (2 years 0 month), developmental delay was not noted. The proband in family 1543 was 1 year 10 months old at the time of genetic test. No clinical features other than hearing loss were notified. Two de novo variants of *SOX10* were identified in sporadic cases in families 1583 and 1651. The c.570C>A (p.Cys190Ter) variant maps to exon 3, and the transcript is predicted to be degenerated by nonsense-mediated decay (NMD) [37], whereas the other variant, c.1122del (p.Thr375ProfsTer127), maps to the last exon (exon 4) and is predicted to escape NMD. No neurologic disorders were recorded in the proband of family 1583, with the c.570C>A variant, whereas the neurologic symptoms of the proband of family 1651, with c.1122del, were consistent with Waardenburg syndrome, with neurological phenotypes (peripheral demyelinating neuropathy, central dysmyelination) associated with escape from NMD [38]. A heterozygous c.1082G>A (p.Arg361Gln) variant of *EYA1*, a gene responsible for Branchiootorenal syndrome 1 (BOR1), was identified in family 1636. The proband with the variant had amblyopia with refractive errors, which have not previously been reported in BOR1, while his father with the heterozygous p.Arg361Gln variant showed mild hearing loss without additional noticeable symptoms. The proband’s mother with normal hearing did not have the variant, and no other family members showed hearing loss. Compound heterozygous variants of *ZNF335* were identified in the proband of family 1456. The two variants were both predicted to affect the region encoding the C2H2-type zinc finger domain; the genetic and clinical features of this family have been reported by others [39].

### Assessment of homogenous large deletion spanning *STRC*

**Fig. 2:**
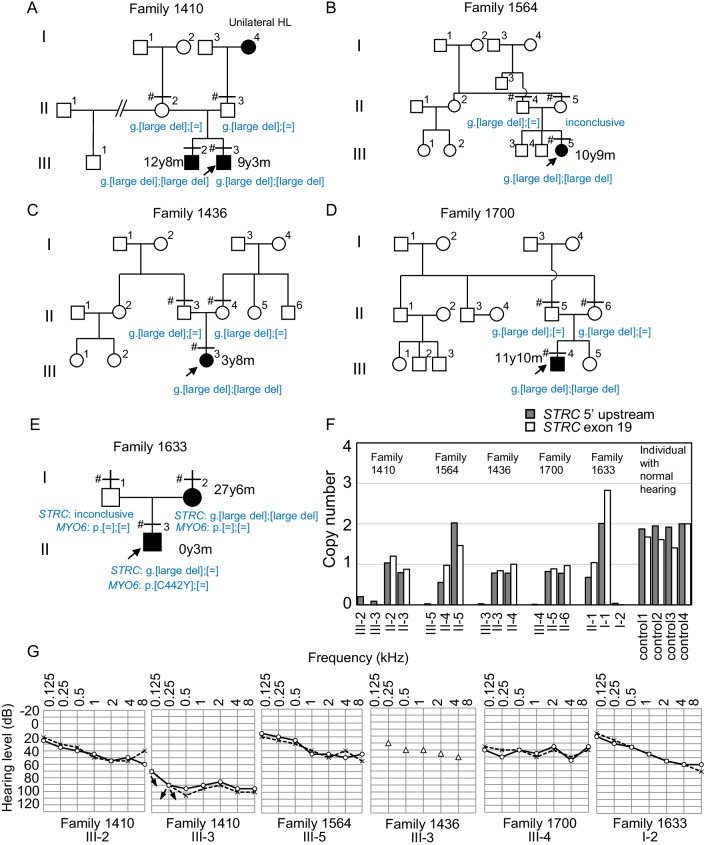
Clinical and genetic features of five families carrying an *STRC* copy number variant*.* **A**–**E** Pedigrees of families with genotype variants in *STRC* or *MYO6*. Horizontal bars with or without sharp symbols (#) above each individual indicate that genotypes were determined by WES and qPCR or qPCR, respectively. Patient genotypes are indicated in blue. **F**, Estimated copy numbers of *STRC* exon 19 and 5′ UTR determined by qPCR. Results from four individuals with normal hearing are shown as controls. **G** Audiograms from patients with homozygous large deletions of the *STRC* region. Open circles and ⨉ symbols indicate hearing level thresholds in the right and left ears, respectively. Open triangles indicate thresholds measured in both ears. Left and right downward arrows indicate that the hearing level was below the indicated level at the respective sound frequency in right and left ears, respectively

While detection of copy number variants (CNVs) from the results of WES is challenging using a single program [40], inspection using the Integrative Genomics Viewer (IGV) suggested that probands in families 1410, 1564, 1436, and 1700, and the mother (I-2) from family 1633, showed extremely low read depths across exon 16 and from exons 19 to 26 of *STRC*, in contrast to a control (III-1 of family 1470), with similar read depths covering all *STRC* exons (Fig. 2A–E, Additional file 7B). Moreover, this large homozygous deleted region appeared to extend to the adjacent gene (exons 8–10 of *CKMT1B*), as well as the entire *CATSPER2* locus, in all probands (Additional file 7C). Homozygous deletion of both *STRC* and *CATSPER2* has been reported to be associated with deafness-infertility syndrome (OMIM: 61102). The non-reduced read depths at other exons (including exons 1–15 and 27–29 of *STRC*, and exon 8 of *CATSPER2*) were likely due to multiple mapping of the sequences of the highly homologous pseudogenes, *STRCP1* and *CATSPER2P1* (Additional file 7D, E) [28, 41].

Multiplex ligation-dependent probe amplification (MLPA) analysis of the probands from families 1410 and 1700 demonstrated homozygous deletion of a genomic region spanning from exon 8 of *CKMT1B* to exon 1 of *CATSPER2* (Additional file 8). According to the positions of the MLPA probes, the 5′ breakpoint was predicted to be between exon 27 of *PPIP5K1* (NC_000015.10:g.43851168) and exon 8 of *CKMT1B* (g.43890333), whereas the 3′ breakpoint was mapped between exon 1 of *CATSPER2* (g.43940784) and exon 1 of *PDIA3* (g.44038794). Based on the inner and outer boundaries, the deleted region was estimated to be between 50.5 and 187.6 kb. This structural variant resembled those recorded in dbVar (for example, nsv868983 (62.4 kb) and nsv3109791 (145.9 kb)) [42]. qPCR targeting of exon 19 and the 5′ UTR of *STRC* also demonstrated absence of these regions of *STRC* in patients from families 1410, 1564, 1436, and 1700 (Fig. 2F), consistent with the results of IGV and MLPA analyses (Fig. 2F, Additional file 7B and Additional file 8). In addition, heterozygous large deletion of an *STRC* allele in the parents of families 1410, 1436, and 1700 was also detected by qPCR (Fig. 2F); however, the copy numbers in the mother (II-5) of family 1564 were difficult to measure, making the exact genotypes predicted to carry the large *STRC* deletion allele ambiguous. The proband (III-3) and a sibling (III-2) in family 1410 had vision loss, in addition to hearing loss.

Intriguingly, IGV predicted homozygous deletion of *STRC* in the mother (I-2) of family 1633 (Additional file 7B), a family initially presumed to have an AD mode of inheritance (Fig. 2E). qPCR demonstrated that the mother (I-2) and the proband (II-1) had homozygous and heterozygous deletion of *STRC*, respectively, whereas the father (I-1) did not appear to have copy number loss of this gene. The trio of family 1633 was reanalyzed under the assumption that a distinct gene was responsible for hearing loss in the proband. Consequently, a de novo variant of *MYO6* (c.1325G>A (p.Cys442Tyr) was identified in the proband.

Because *OTOA* is also known to have highly homologous pseudogene *OTOAP1* especially in its exon 21–29, we searched for differences in read depths of *OTOA*. However, we could not detect any changes suggesting large deletion or duplication of *OTOA* in any probands.

### Novel candidate genes associated with hearing loss

**Fig. 3:**
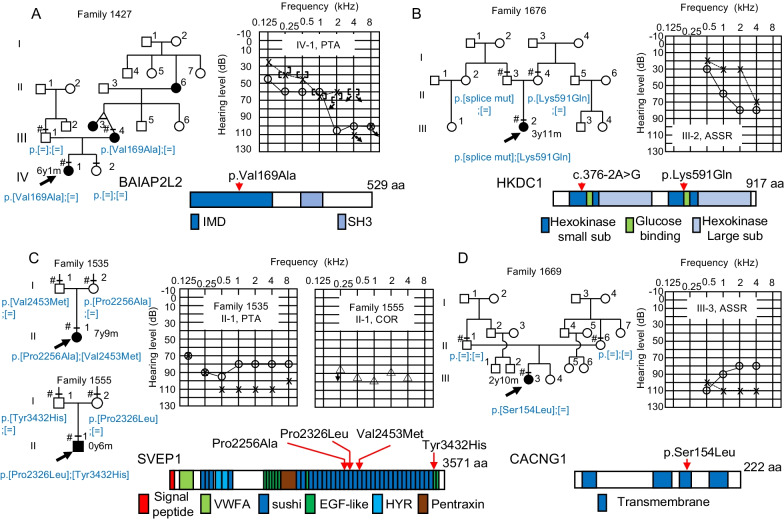
Pedigrees with novel candidate genes associated with hearing loss. **A** *BAIAP2L2*; **B** *HKDC1*; **C** *SVEP*1; **D** *CACNG1*. Genotypes of candidate genes are shown in the pedigree. Audiograms of the probands, and predicted structures of gene products are shown. Horizontal bars with or without sharp symbols (#) above each individual indicate that genotypes were determined by WES and Sanger sequencing or Sanger sequencing, respectively. ASSR, auditory steady-state response; COR, conditioned orientation response; PTA, pure-tone audiometry. Positions of each candidate variant on the primary structure of the gene product are also indicated, along with the structural or functional domains of the product

**Fig. 4:**
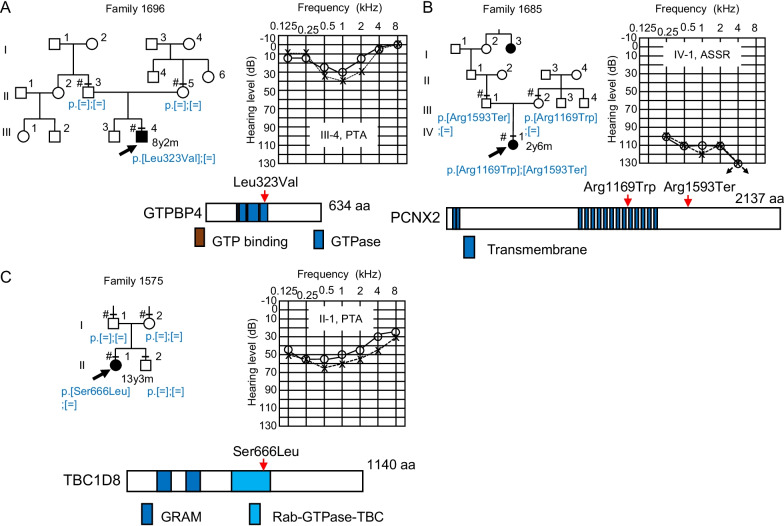
Pedigrees with novel candidate genes associated with hearing loss (continued from Fig. 3). **A** *GTPBP4*; **B** *PCNX2*; **C** *TBC1D8*. Genotypes of candidate genes are shown in the pedigree. Audiograms of the probands, and positions of each candidate variant on the primary structure of the gene product are also indicated, along with the structural or functional domains of the product

In addition to the previously known deafness genes categorized to Tier 1, eight additional genes were narrowed down as single candidates by WES analysis in a total of 10 families (Figs. 1, 3, 4). Two of these genes (*SLC12A2* and *BAIAP2L2*, Tier 2) cause hearing loss phenotypes in mouse models, and one (*HKDC1*, Tier 3) is predominantly expressed in *Macaca fascicularis* cochlea. The other five genes (*SVEP1*, *CACNG1*, *GTPBP4*, *PCNX2*, and *TBC1D8*) were categorized as Tier 4 genes, with no known association with hearing loss. Genetic information for each variant is presented in Additional file 9. Partial Sanger sequencing electropherograms validating each variant are presented in Additional file 10. Association of *SLC12A2* variants with hearing loss has been reported [12] and registered as DFNA78 in OMIM (619081).

A heterozygous variant of *BAIAP2L2* was identified as the candidate cause for AD inheritance mode hearing loss in family 1427 (Fig. 3A). This gene encodes the membrane protein, brain-specific angiogenesis inhibitor 1-associated protein 2-like protein 2, which localizes to the plasma membrane in intestine and kidney epithelial cells [43]. Further, single-cell RNA sequencing analysis demonstrated predominant *Baiap2l2* expression in hair cells in neonatal mouse cochlear epithelium [44] (Additional file 11), and mice deficient for *Baiap2l2* have an increased auditory brainstem response threshold [24, 45]. The c.506T>C (p.Val169Ala) variant is predicted to reside in the IRSp53/MIM homology domain (IMD), which can bind to membranes and interact with a small GTPase (PROSITE: PRU00668) [46]. The proband with heterozygous *BAIAP2L2* variant showed congenital, progressive, severe, steep sloping hearing loss without other symptoms.

Compound heterozygous *HKDC1* variants were identified as the candidate cause of the sporadic hearing loss in family 1676 (Fig. 3B). This gene encodes hexokinase domain-containing 1, which catalyzes phosphorylation of glucose to generate glucose-6-phosphate [47]. A genome-wide association study (GWAS) identified *HKDC1* as a risk factor for gestational hyperglycemia [48]. The missense variant found in the proband (c.1771A>C (p.Lys591Gln)) was predicted to reside in the hexokinase small subdomain 2, whereas the other compound heterozygous variant was predicted to affect splicing (c.376–2A>G). The proband had congenital, mild-to-moderate hearing loss, without other symptoms.

Compound heterozygous variants of *SVEP1* were identified as the candidate cause of the sporadic hearing loss in two families: 1535 and 1555 (Fig. 3C). This gene encodes Sushi von Willebrand factor type A EGF and pentraxin domain-containing 1, which may function in cell attachment via integrin α9β1 [49]. A GWAS detected *SVEP1* as a risk factor for coronary artery disease [50] and knockout of *Svep1* in mice is embryonic lethal, with multiple developmental defects [25]. All four variants (c.6766C>G (p.Pro2256Ala), c.7357G>A (p.Val2453Met), c.6977C>T (p.Pro2326Leu), and c.10294T>C (p.Tyr3432His)) found in this study reside in the stretched sushi domains. The probands in families 1535 and 1555 carried the compound heterozygous variants c.[6766C>G];[7357G>A] and c.[6977C>T];[10294T>C], respectively, and had congenital, severe-to-profound non-syndromic hearing loss, without other symptoms.

A de novo heterozygous variant of *CACNG1* was identified as the candidate cause of the sporadic hearing loss in family 1669 (Fig. 3D). This gene encodes voltage-dependent calcium channel gamma-1 subunit. The c.461C>T (p.Ser154Leu) variant of *CACNG1* is predicted to encode a residue in the transmembrane domain of the putative protein product. *Cacng1*-knockout mice show dysregulated calcium transport in skeletal muscle [51]. The proband with the variant showed congenital, severe-to-profound hearing loss, without other symptoms.

A de novo heterozygous variant of *GTPBP4* was identified as the candidate cause of the sporadic hearing loss in family 1696 (Fig. 4A). This gene encodes a nucleolar GTP-binding protein, and the variant (c.967C>G (p.Leu323Val)) in this gene affects the predicted GTP-binding domain. GTPBP4 mediates ribosomal RNA processing [52], suppresses schwannoma cell growth [53], and promotes colorectal carcinoma metastasis [54] in vitro. The proband (III-4) with the variant showed congenital, mild, and mid-frequency hearing loss, without other symptoms.

Compound heterozygous variants of *PCNX2* were identified as the candidate cause of the AR inheritance mode hearing loss in family 1685 (Fig. 4B). This gene encodes Pecanex-like protein 2 and is frequently mutated in colorectal carcinomas with high microsatellite instability [55]. The detected variants were a nonsense change (c.4777C>T (p.Arg1593Ter)) and a missense variant (c.3505C>T (p.Arg1169Trp)), residing in the intracellular region of the plasma membrane protein. *Pcnx2* deficiency modifies seizure-like behaviors in mouse [56]. The proband had congenital, progressive hearing loss, resulting in profound hearing loss at 2 years old, as well as abnormal pulmonary venous return, which was surgically treated at 1 day after birth.

A de novo heterozygous variant of *TBC1D8* was identified as a candidate cause of the sporadic hearing loss in family 1575 (Fig. 4C). This gene encodes Tre-2 BUB2p and Cdc16p domain 1 family member 8, which functions as a GTPase-activator of Rab family proteins and promotes tumorigenesis of ovarian cancer [57]. *TBC1D8* has also been reported to be within a susceptibility locus for osteoporosis-related traits [58]. The variant c.1997C>T (p.Ser666Leu) was predicted to reside in the putative carboxyl-terminal Rab-GTPase-TBC domain with unknown function. The proband with the variant had congenital, moderate low-frequency hearing loss, without other symptoms.

## Discussion

### Identification of variants in known deafness genes by WES analysis

Analysis of Tier 1 prioritized genes using WES data led to successful identification of pathogenic or likely pathogenic variants in 11 known deafness genes in 21 of 72 families, after screening of common deafness genes. Due to higher coverage of coding regions, WES is considered to detect pathogenic variants more efficiently and more cost-effectively than WGS. In addition, we narrowed down eight single genes as candidates associated with hearing loss in 10 families. Analysis of prioritized Tier 1 genes was similarly effective to targeted NGS analysis [3] and enabled efficient determination of the genes responsible for hearing loss in probands. After prescreening for *GJB2,* m.1555A>G, and m.3243A>G variants, as well as *SLC26A4* and *OTOF* variants, when patient data suggested, the two most frequently identified genes in this study were *STRC* (DFNB16, five families) and *MYO15A* (DFNB3, four families). These two genes have been reported as relatively frequent causes of genetic hearing loss in Japan [59, 60] and studies in other ethnic regions [4, 61]. Subsequently, *CDH23*, *PDZD7*, and *PTPN11* were detected as causative genes in two families each. Unlike *CDH23* and *PDZD7*, which cause non-syndromic hearing loss or Usher syndrome presenting as non-syndromic hearing loss during childhood, *PTPN11* is associated with NS1, which shows a variety of phenotypes in multiple organs [5]. Although two probands with *PTPN11* variants had short stature, and one exhibited café-au-lait pigmentation, these clinical features had been unnoticed by the primary physicians. Our findings highlight that NS1 with no-to-mild symptoms, other than hearing loss, can be categorized as non-syndromic hearing loss in certain cases; hence *PTPN11* may be a much more frequent cause of hearing loss than previously recognized.

Although a straightforward method to detect CNVs from WES data has yet to be established, homozygous deletion of *STRC*, which harbors a tandem homologous pseudogene sequence at its genomic locus, with potential for non-allelic homologous recombination [62], was successfully detected by combined assessment of read depths for each coding exon, MLPA, and qPCR. More extensive analyses of structural variants using several programs [63, 64], WGS [65], and long read sequencing [66] would reveal exact breakpoints of *STRC* CNVs.

In addition, this study demonstrated that trio WES analysis is a potent method of deciphering the reasons for discrepancies between pedigree and genetic inheritance, as shown in family 1633, where there was an initial presumption of AD inheritance, but mutations at two separate loci (*STRC* and *MYO6*) were detected. This study also demonstrates that WES analysis can be used to identify genes responsible for hearing loss and other factors suspected of influencing coexisting symptoms, to explain the clinical features in families; for example, families 1636 (*EYA1* variant with amblyopia) and 1410 (*STRC* variant with vision loss).

### Strategy to discover novel candidate deafness genes by WES analysis

Our strategy to discover novel candidate genes associated with hearing loss from Tiers 2–4 genes was based on the assumption that deafness genes would also cause auditory phenotypes in animal models [24, 25], which we categorized as Tier 2 genes, and that many genes critical for proper hearing in humans would also show predominant expression in *M. fascicularis* cochlea [26], which we categorized as Tier 3 genes. We identified *SLC12A2*, *BAIAPL2*, and *HKDC1* as promising candidate genes warranting investigation for pathogenicity; however, identification of additional patients with variants in the same candidate genes will be critical for confirming their involvement. *SVEP1* variants were detected in two families and are plausible candidates for further investigation, such as in vitro functional analysis or generation of an animal model with the identified variants knocked in. Confirmation of novel deafness genes will improve genetic tests for hearing loss.

We were unable to screen single candidate genes in 35 families, and no candidate variants emerged from WES analysis in six families. Hearing loss in these families may be attributable to pathogenic variants in untranslated regions, introns, cryptic splice sites, promoter or enhancer regions, intergenic regions, multigenic causes, or chromosomal arrangements, including CNVs, or unidentified environmental factors. We also aware that 5 exonic regions n Tier 1 genes showed insufficient read depth. Variants on these exons may also have been failed to be detected. In addition, our in silico filtering strategy did not use REVEL scores recommended by Hearing Loss Expert Panel guidelines [67]. In fact, our filtering strategy is considered very stringent; variants were filtered out only when all the in silico analyses (see “Methods” section) predicted no, benign, or tolerated effect. As a result, two candidate variants on our list showed low REVEL scores (*PDZD7*:c.503G>C, REVEL = 0.123, (Table 3) and *PCNX2*:c.3505C>G, REVEL = 0.139 (Additional file 9)). Although we cannot exclude out the possibility of filtering out pathogenic variants based on in silico prediction, it is considered quite unlikely.

Another possibility is that we may have missed causative genes due to discrepancies between the typical clinical features caused by the gene and those observed in our probands. For example, Tier1 genes included *KDM6A,* a gene responsible for syndromic hearing loss (Kabuki syndrome 2; OMIM: 300827). Variants of this gene were not considered as candidates when the proband had non-syndromic hearing loss; however, we cannot exclude the possibility that these variants can be associated with very mild or normal phenotypes, except for hearing loss. As we experienced in the case with known pathogenic variant of *PTPN11* in family 1543, clinical features of several diseases such as Noonan syndrome show wide spectrum of symptoms including non-syndromic hearing loss, and these atypical features in patients could have been overlooked and affected the diagnostic yield. It is also possible that symptoms other than hearing loss are late-onset and overlooked at the time of genetic test. These are the limitations of this study to detect Tier 1 genes associated with hearing loss using WES analysis.

## Conclusions

WES analysis using a tier system to prioritize genetic analysis is an efficient method to identify pathogenic variants of known deafness genes, as well as novel candidate deafness genes. Further analyses, including accumulation of variants and clinical features of patients, will expand perspectives on hereditary hearing loss.

## Supplementary Information

**Additional file 1.** Summary of whole exome sequencing results.

**Additional file 2.** Flowchart of WES analysis. All detected variants affecting protein-coding sequences with low minor allele frequencies (MAF) in global and Japanese populations were subjected to further filtration. Variants were categorized in four tiers of genes and subjected to co-segregation analysis. See “Materials and methods” for details.

**Additional file 3.** List of genes categorized in Tier 3 in this study.

**Additional file 4.** List of captured regions with insufficient average read depths (<20) of Tier 1 genes in this study.

**Additional file 5.** Primers used in this study.

**Additional file 6.** Partial electropherograms of variants in known deafness genes detected in this study. Green, blue, black, and red peaks indicate nucleotides **A**, **C**, **G**, and **T**, respectively. Data were derived from probands from: (**A**) and (**B**), family 1470; (**C**), family 1540; (**D**) and (**E**), family 1479; (**F**), family 1688; (**G**) and (**H**), family 1644; (**I**), family 1528; (**J**) and (**K**), family 1397; (**L**), family 1597; (**M**), family 1648; (**N**) and (**O**), family 739; (**P**), family 1633; (**Q**), family 1543; (**R**), family 1631; (**S**), family 1583; (**T**), family 1651; (**U**), family 1636; (**V**) and (**W**), family 1456. Reverse complementary sequences are shown in (**F**), (**M**), (**O**), (**R**), and (**T**). Segregation of all variants in probands and their parents was validated by Sanger sequencing. Note that in (**F**), c.8969delG variant of *MYO15A* is based on right-normalized nomenclature and not c.8968-1delG as shown by electropherogram.

**Additional file 7.** Genome map of the *STRC *locus and a homozygous large deletion of *STRC* and *CATSPER2* visualized using Integrative Genomics Viewer (IGV). (**A**), Partial chromosomal 15q15.3 locus visualized using IGV. Genes are shown in blue. (**B**) and (**C**), Representative IGV images of WES reads mapped to *CKMT1B*, *STRC *(**B**), and *CATSPER2 *(**C**) in probands from families 1410, 1564, 1436, and 1700, and I-2 from family 1633. WES reads in the proband of family 1470 are shown as a control to represent normally mapped reads in the locus. Positions of exons examined by MLPA or mentioned in the manuscript are indicated with arrows. (**D**) and (**E**), Multiple mapped reads (blank boxes) at, for example, the exon 1–15 and exon 27–29 regions of *STRC*, due to inability to distinguish sequences from *STRC* and *STRCP1* (**D**), and exon 8 of *CATSPER2* due to inability to distinguish sequences from *CATSPER2* and *CATSPER2P1* (**E**). Single mapped reads are shown in gray boxes.

**Additional file 8.** Homozygous large deletion of the locus containing *STRC* and* CATSPER2* detected by multiplex ligation-dependent probe amplification (MLPA). Representative MLPA results showing homozygous deletion of the region including the partial *CKMT1B* and entire *STRC* and *CATSPER2* genes in the probands from families 1410 and 1700. Estimated copy numbers of each exon are shown as mean ± S.D.

**Additional file 9.** Variants of novel candidate genes associated with hearing loss.

**Additional file 10.** Electropherograms showing variants in novel candidate genes associated with hearing loss. Data are derived from probands from (**A**), family 1427; (**B**) and (**C**), family 1676; (**D**) and (**E**), family 1535; (**F**) and (**G**), family 1555; (**H**), family 1669; (**I**), family 1696; (**J**) and (**K**), family 1685; and (**L**), family 1575. Reverse complementary sequences are shown in (**C**), (**K**), and (**L**).

**Additional file 11.** Predominant expression of *Baiap2l2 *in auditory hair cell clusters. Images are derived from single-cell RNA sequencing analysis of mouse cochlear epithelium at postnatal day 1 from gEAR portal (https://umgear.org). For detailed classification of the cell clusters, see Kolla et al. (2020). DC, Deiter’s cells row 1–3; Hensen, Hensen’s cells; IHC, inner hair cells; IPC, inner pillar cells; IPhC, inner phalangeal cells/border cells; IS, inner sulcus cells; IdC, interdental cells; LGER, lateral greater epithelial ridge cells group 1–3; MGER, medial greater epithelial ridge cells; OHC, outer hair cells; OPC, outer pillar cells; OS, outer sulcus cells; Oc90, Oc90-positive cells; eIHC, less mature developing inner hair cells; eOHC, less mature developing outer hair cells.

## Data Availability

The ethics committee approves sharing filtered and limited number of variants detected from each subject to public, but does not approve sequencing data of each individual open to public. All the pathogenic or candidate pathogenic variants detected from are within the manuscript and its Additional files.

## Abbreviations

WES: Whole exome sequencing
CNV: Copy number variant
