# Supplementary material for: Whole exome analysis of patients in Japan with hearing loss reveals high heterogeneity among responsible and novel candidate genes
Source: Orphanet J Rare Dis. 2022 Mar 5;17:114. doi: 10.1186/s13023-022-02262-4 (PMC8898489; doi:10.1186/s13023-022-02262-4)
Supplement: Supplementary file 1 — Additional file 1. Summary of whole exome sequencing results. [file 13023_2022_2262_MOESM1_ESM.pdf]

Additional file 1. Summary of whole exome sequencing results.

Category	Value
Average mapping rate	0.999184 ± 0.000252
Average duplication rate	0.092579 ± 0.040129
Average depth	144.425984 ± 41.984708
Average number of SNVs	78599.42 ± 1006.318316
Average number of indels	12736.013333 ± 649.132927
