# Supplementary figures and images for: Whole exome analysis of patients in Japan with hearing loss reveals high heterogeneity among responsible and novel candidate genes
Source: Orphanet J Rare Dis. 2022 Mar 5;17:114. doi: 10.1186/s13023-022-02262-4 (PMC8898489; doi:10.1186/s13023-022-02262-4)

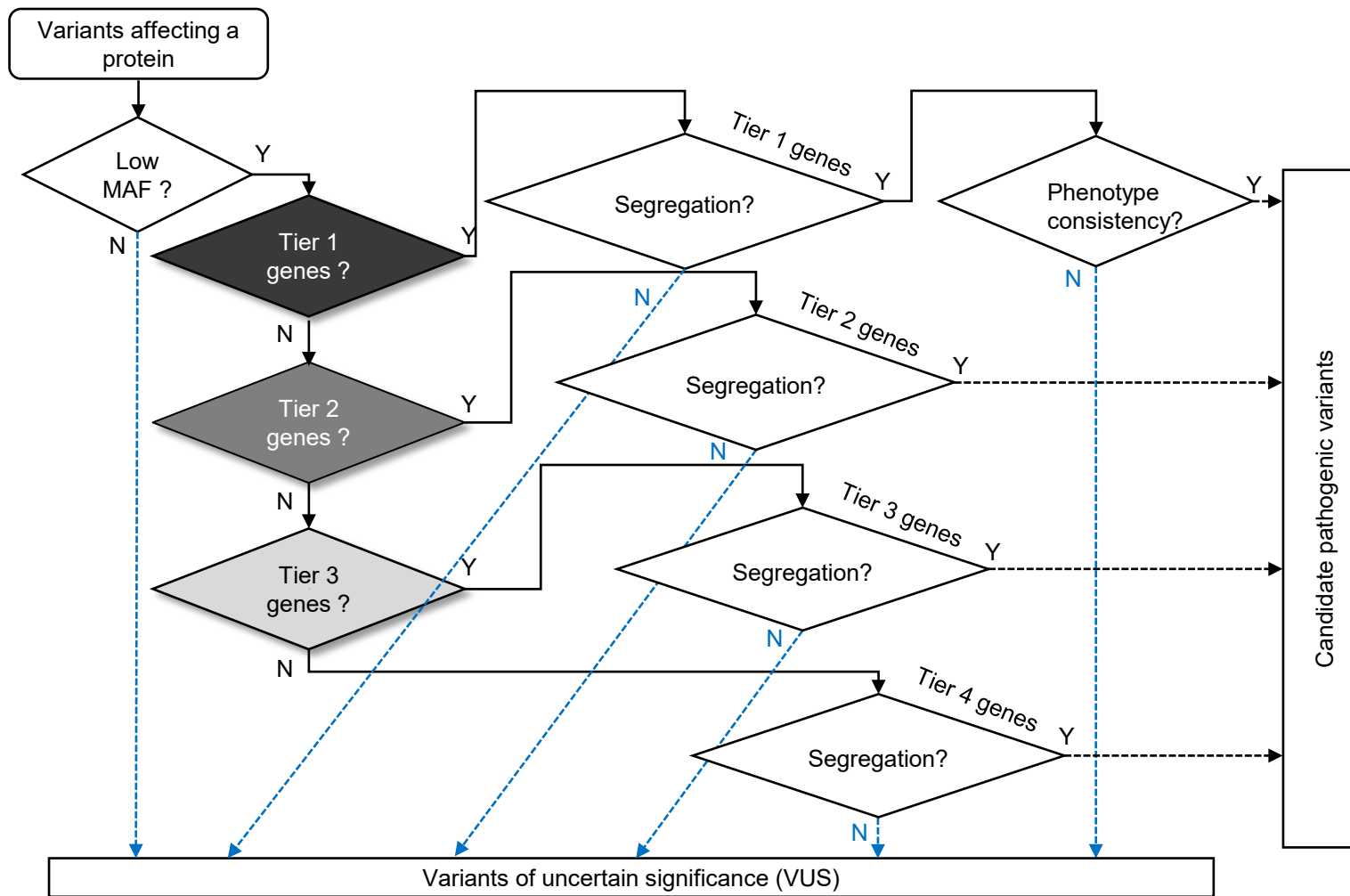

Supplement: Supplementary file 2 — Additional file 2. Flowchart of WES analysis. All detected variants affecting protein-coding sequences with low minor allele frequencies (MAF) in global and Japanese populations were subjected to further filtration. Variants were categorized in four tiers of genes and subjected to co-segregation analysis. See “Materials and methods” for details. [file 13023_2022_2262_MOESM2_ESM.pdf]

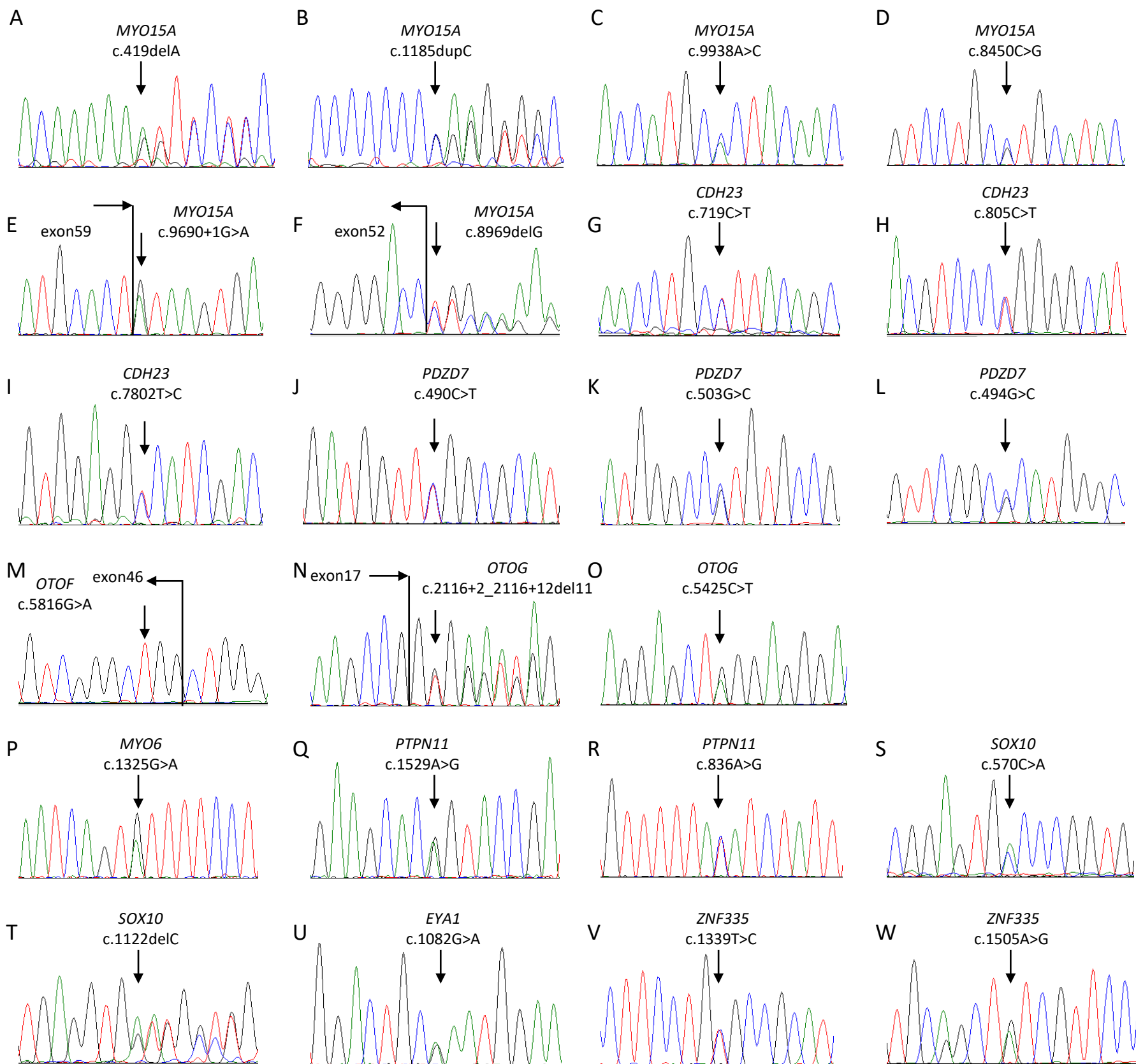

Supplement: Supplementary file 6 — Additional file 6. Partial electropherograms of variants in known deafness genes detected in this study. Green, blue, black, and red peaks indicate nucleotides A, C, G, and T, respectively. Data were derived from probands from: (A) and (B), family 1470; (C), family 1540; (D) and (E), family 1479; (F), family 1688; (G) and (H), family 1644; (I), family 1528; (J) and (K), family 1397; (L), family 1597; (M), family 1648; (N) and (O), family 739; (P), family 1633; (Q), family 1543; (R), family 1631; (S), family 1583; (T), family 1651; (U), family 1636; (V) and (W), family 1456. Reverse complementary sequences are shown in (F), (M), (O), (R), and (T). Segregation of all variants in probands and their parents was validated by Sanger sequencing. Note that in (F), c.8969delG variant of MYO15A is based on right-normalized nomenclature and not c.8968-1delG as shown by electropherogram. [file 13023_2022_2262_MOESM6_ESM.pdf]

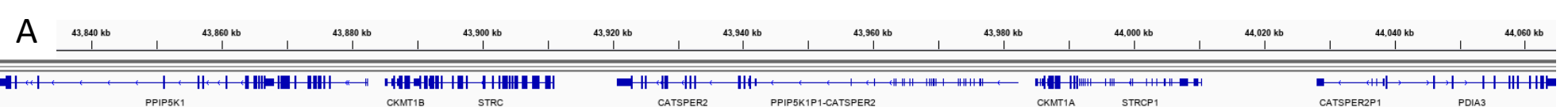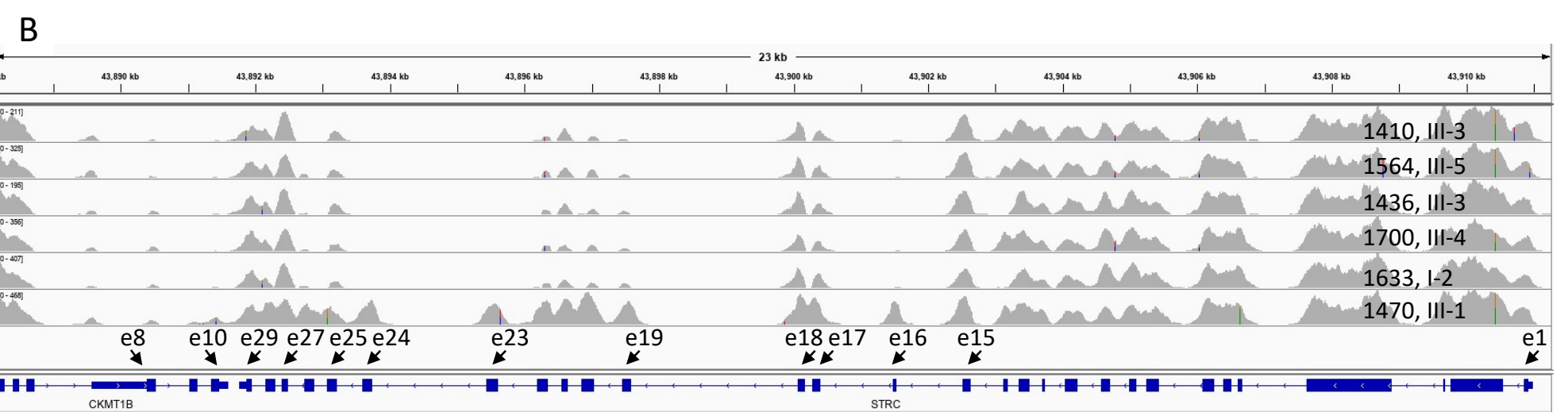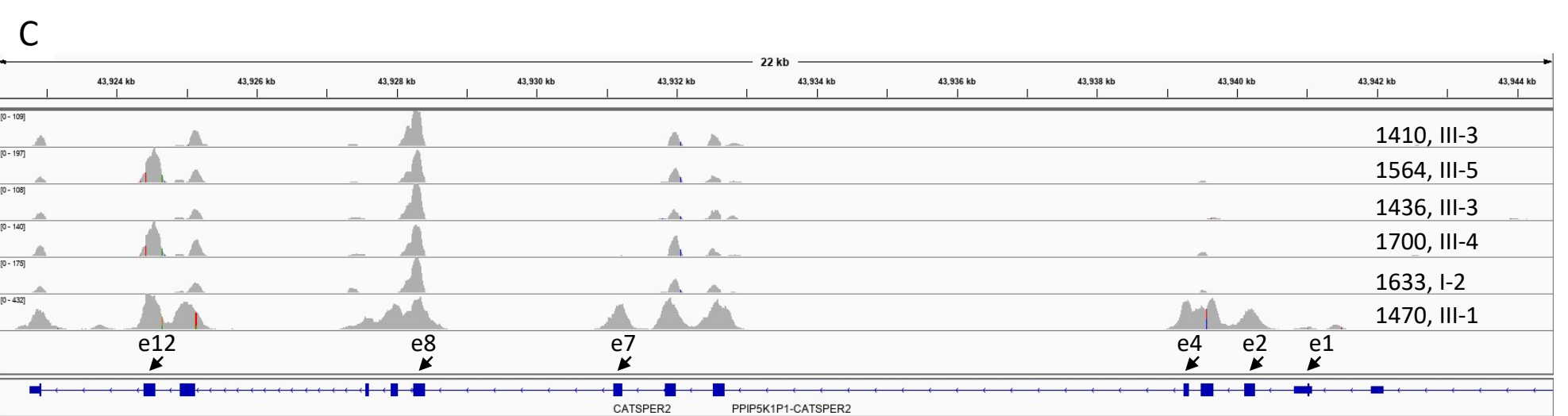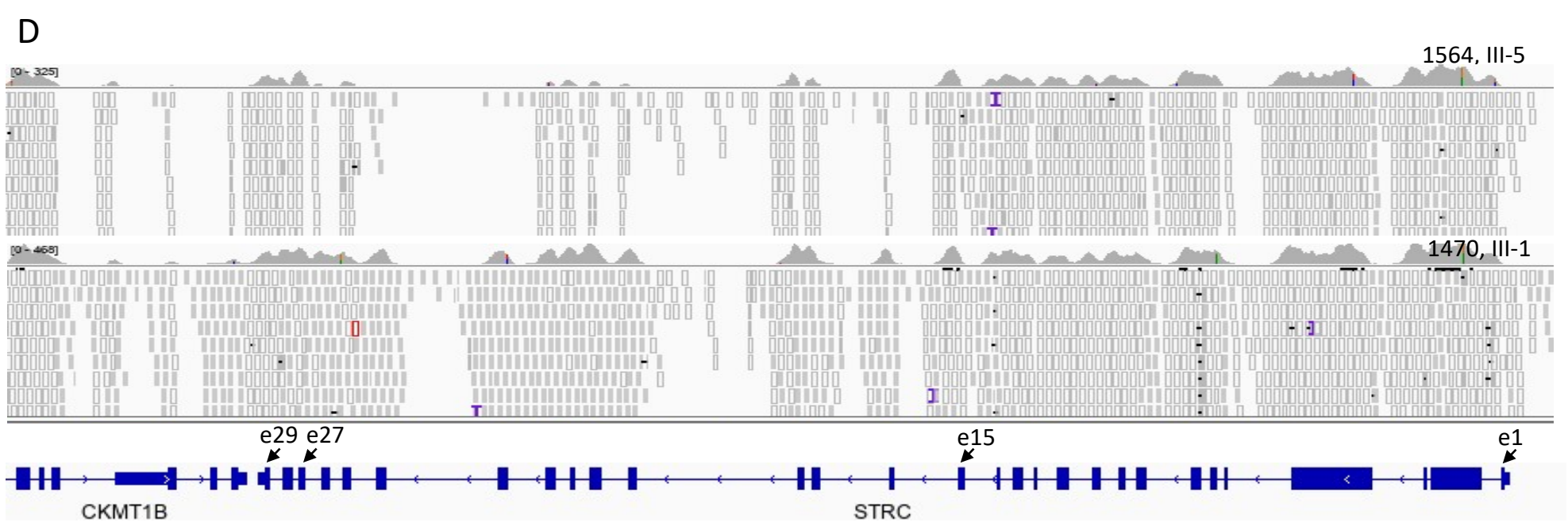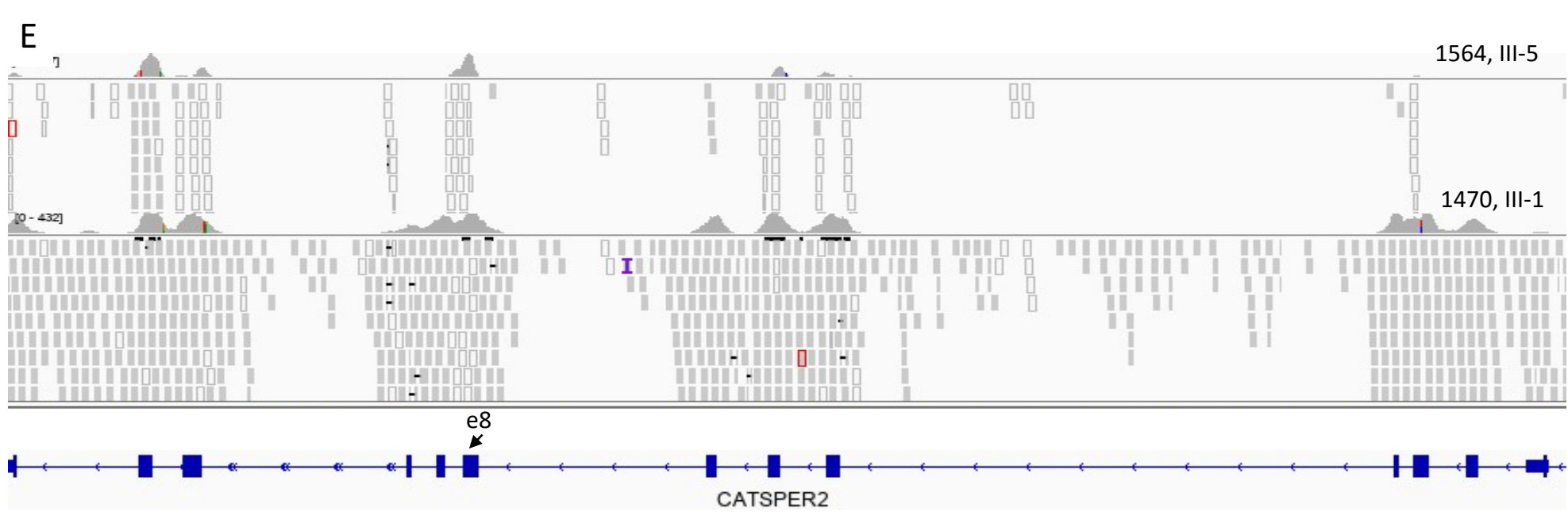

Supplement: Supplementary file 7 — Additional file 7. Genome map of the STRC locus and a homozygous large deletion of STRC and CATSPER2 visualized using Integrative Genomics Viewer (IGV). (A), Partial chromosomal 15q15.3 locus visualized using IGV. Genes are shown in blue. (B) and (C), Representative IGV images of WES reads mapped to CKMT1B, STRC (B), and CATSPER2 (C) in probands from families 1410, 1564, 1436, and 1700, and I-2 from family 1633. WES reads in the proband of family 1470 are shown as a control to represent normally mapped reads in the locus. Positions of exons examined by MLPA or mentioned in the manuscript are indicated with arrows. (D) and (E), Multiple mapped reads (blank boxes) at, for example, the exon 1–15 and exon 27–29 regions of STRC, due to inability to distinguish sequences from STRC and STRCP1 (D), and exon 8 of CATSPER2 due to inability to distinguish sequences from CATSPER2 and CATSPER2P1 (E). Single mapped reads are shown in gray boxes. [file 13023_2022_2262_MOESM7_ESM.pdf]

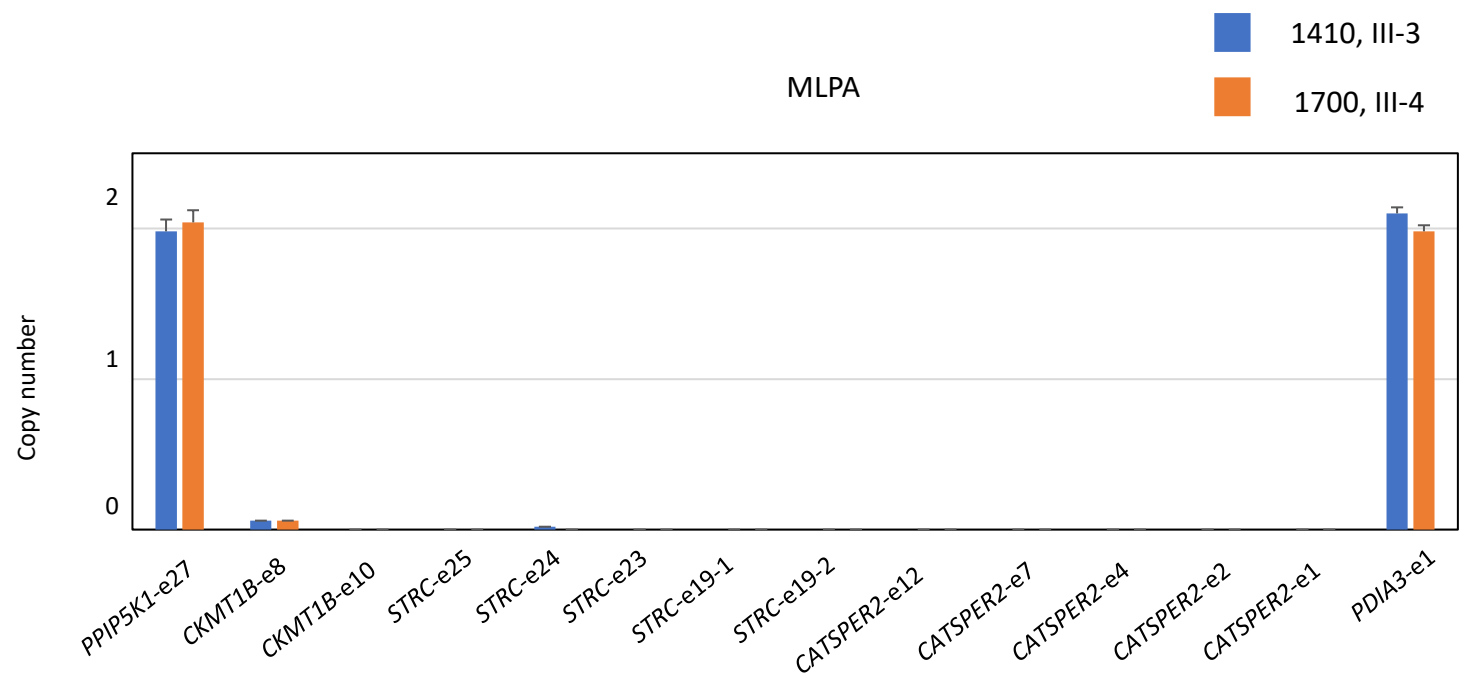

Supplement: Supplementary file 8 — Additional file 8. Homozygous large deletion of the locus containing STRC and CATSPER2 detected by multiplex ligation-dependent probe amplification (MLPA). Representative MLPA results showing homozygous deletion of the region including the partial CKMT1B and entire STRC and CATSPER2 genes in the probands from families 1410 and 1700. Estimated copy numbers of each exon are shown as mean ± S.D. [file 13023_2022_2262_MOESM8_ESM.pdf]

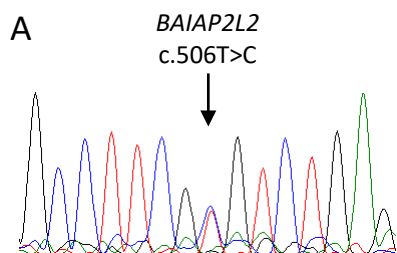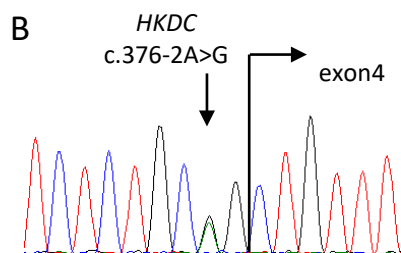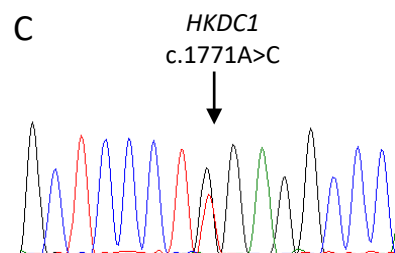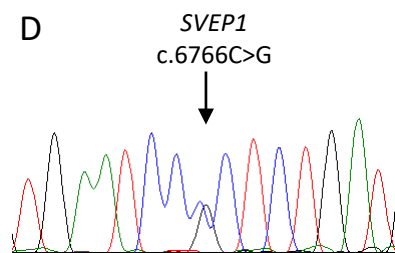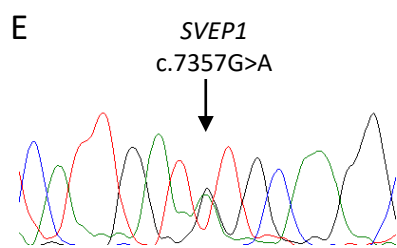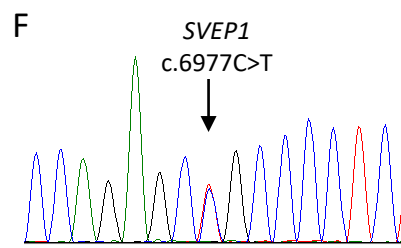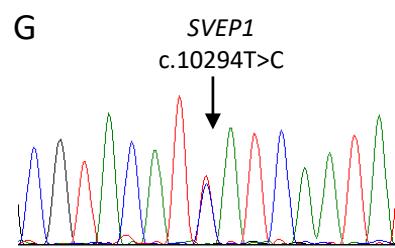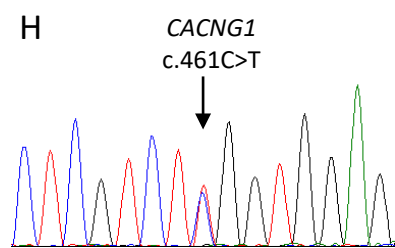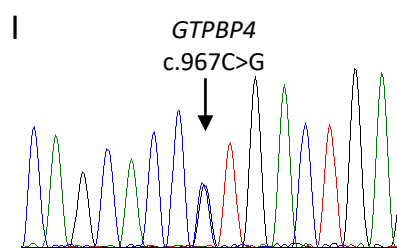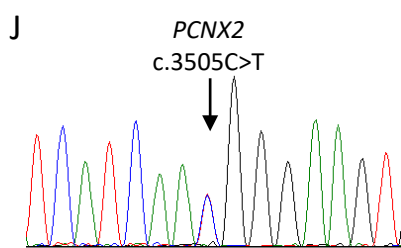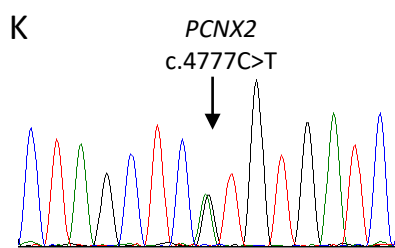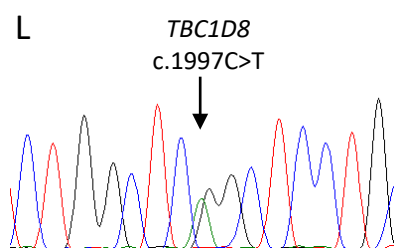

Supplement: Supplementary file 10 — Additional file 10. Electropherograms showing variants in novel candidate genes associated with hearing loss. Data are derived from probands from (A), family 1427; (B) and (C), family 1676; (D) and (E), family 1535; (F) and (G), family 1555; (H), family 1669; (I), family 1696; (J) and (K), family 1685; and (L), family 1575. Reverse complementary sequences are shown in (C), (K), and (L). [file 13023_2022_2262_MOESM10_ESM.pdf]

Baiap2l2

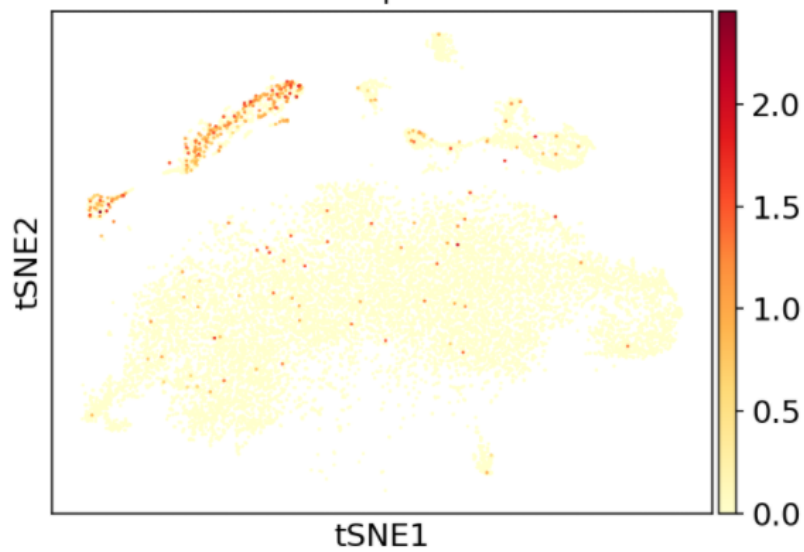

cell\_type

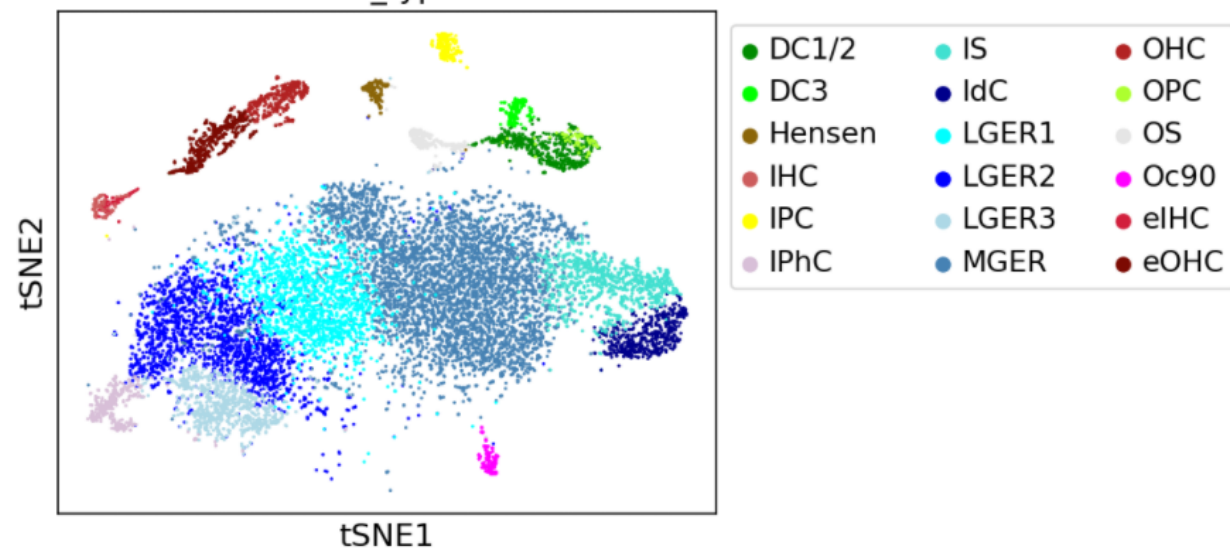

Supplement: Supplementary file 11 — Additional file 11. Predominant expression of Baiap2l2 in auditory hair cell clusters. Images are derived from single-cell RNA sequencing analysis of mouse cochlear epithelium at postnatal day 1 from gEAR portal (https://umgear.org). For detailed classification of the cell clusters, see Kolla et al. (2020). DC, Deiter’s cells row 1–3; Hensen, Hensen’s cells; IHC, inner hair cells; IPC, inner pillar cells; IPhC, inner phalangeal cells/border cells; IS, inner sulcus cells; IdC, interdental cells; LGER, lateral greater epithelial ridge cells group 1–3; MGER, medial greater epithelial ridge cells; OHC, outer hair cells; OPC, outer pillar cells; OS, outer sulcus cells; Oc90, Oc90-positive cells; eIHC, less mature developing inner hair cells; eOHC, less mature developing outer hair cells. [file 13023_2022_2262_MOESM11_ESM.pdf]
