# Supplementary material for: Whole exome analysis of patients in Japan with hearing loss reveals high heterogeneity among responsible and novel candidate genes
Source: Orphanet J Rare Dis. 2022 Mar 5;17:114. doi: 10.1186/s13023-022-02262-4 (PMC8898489; doi:10.1186/s13023-022-02262-4)
Supplement: Supplementary file 3 — Additional file 3. List of genes categorized in Tier 3 in this study. [file 13023_2022_2262_MOESM3_ESM.pdf]

Additional file 3. List of genes categorized in Tier 3 in this study.

MLANA	*MAPK8IP2*	*HKDC1*	*SPAG11*	*IRX6*	*SCCPDH*	*ITSN1*
PSMD12	*DSC1*	*HTRA1*	*ECM2*	*IL18*	*EPM2A*	*USP32*
SLCO2B1	*FMO3*	*CRISP3*	*STX1B*	*HPGD*	*TAB2*	*DST*
S100B	*TRPM1*	*DUT*	*EIF5A2*	*SLCO1B1*	*FNTA*	*PSMA2*
COL10A1	*TMEM213*	*LTBP4*	*SLC25A13*	*HOXD1*	*KRTDAP*	*C15orf40*
TUFM	*ABCA10*	*KRT23*	*WNT16*	*MORF4L1*	*EPYC*	
NEFH	*RDH5*	*ALDH1A3*	*ZFHX3*	*RBMS3*	*FLG2*	
ANO5	*PLLP*	*SPTLC3*	*OR51E2*	*DACH1*	*SLC17A6*	
MLIP	*CA14*	*RDH10*	*ID4*	*LOC696306*	*KCTD4*	
SHC4	*CA13*	*TM4SF18*	*SLC5A3*	*MFAP3L*	*C17orf67*	
SLC22A2	*METTL7B*	*SMCO3*	*PFKFB3*	*FIBIN*	*NRG1*	
PVALB	*ITIH2*	*CTXN3*	*SOX17*	*GRHL1*	*cDNA FLJ43186 fis*	
ZIC2	*PRH2*	*SCN7A*	*CFB*	*TFAP2B*	*LANCL3*	
UGT8	*FILIP1*	*GPR87*	*WFDC5*	*CPSF6*	*PLEKHA4*	
SCEL	*RARRES1*	*ITGB8*	*ABI3BP*	*LOC720403*	*LOC100288310*	
LOC718942	*EBF1*	*CRTAC1*	*LOC702904*	*PIK3R1*	*cDNA IMAGE:1625225*	
SLC27A6	*COL8A2*	*UPK1B*	*GPR137B*	*GAL3ST1*	*LECT1*	
SLCO1A2	*C2ORF40*	*MRAP2*	*SPTBN1*	*WDR11*	*PCP4*	
KRT24	*ASPA*	*WDR18*	*TM7SF2*	*SCUBE2*	*IRX5*	
SCIN	*CES1*	*FIG4*	*MEOX2*	*FREM1*	*POU4F2*	
SV2C	*CHST9*	*STAC*	*COL9A3*	*C1ORF162*	*POU4F1*	
GAS2	*AADACL2*	*SCARA5*	*EGFL8*	*GANC*	*PLCB4*	
SPP1	*ABCA9*	*TNFRSF19*	*ZFHX4*	*UST*	*cDNA FLJ37676 fis*	
DMP1	*MUC15*	*MGP*	*FRMD3*	*ACSL1*	*C12orf69*	
DCLK3	*CRISPLD1*	*BCAS1*	*IL17B*	*GDPD3*	*FLG*	
Mamu_482871	*IPO5*	*OLFM4*	*ANXA3*	*WNK3*	*KCNN2*	
PheRS	*SPATA22*	*CCDC114*	*ARL9*	*ERMP1*	*LRRN1*	
SERPIND1	*IL20RB*	*SVIP*	*TMPRSS11E*	*GPC6*	*MAGI1*	
CLDN8	*CTDSP2*	*MS4A7*	*PROM1*	*CSRP2*	*CARD18*	
DSC2	*DCP2*	*F13A1*	*PLEKHG7*	*IFIT1*	*RAB12*	
VTCN1	*EBF2*	*CST6*	*RPL24*	*MPP6*	*cDNA*	
C19H19orf33	*GFRA1*	*INSC*	*DMKN*	*BGLAP*	*LOC283143*	
LOC717747	*SULF1*	*CDH19*	*UACA*	*MPZL2*	*FAM190A*	
CRYAB	*WDR86*	*RERGL*	*EBF3*	*MTMR6*	*RAD54B*	
LGR5	*MEPE*	*HEY2*	*CPXM2*	*EEF1D*	*GRIK2*	
OVOS	*SFRP4*	*ELOVL7*	*COLEC12*	*NPNT*	*LOC220077*	
IBSP	*LOC693624*	*MALL*	*ABLIM1*	*FAM162A*	*DDR2*	
CP	*ANXA4*	*PPP1R1C*	*B3GNT5*	*CD55*	*NFIB*	
PAPSS2	*KIAA1024*	*PROS1*	*GSN*	*GTPBP8*	*AKAP12*	
P2RY2	*TNNT1*	*HHATL*	*CDK2*	*RORA*	*ITGA10*	
KLK7	*KIF21A*	*CALML5*	*LXN*	*TMCC3*	*CADM1*	
CLCA2	*OSMR*	*LOC693471*	*MEGF10*	*NEK1*	*TMEM117*	
OGN	*MS4A6A*	*LDLRAD3*	*MRPS26*	*NT5DC1*	*RARB*	
DEFB122	*UBA6*	*EGR2*	*LASS3*	*GSTM4*	*PLAG1*	
OMD	*CRABP1*	*PTPRU*	*CYP26A1*	*DLGAP5*	*SECISBP2L*	
SLC13A4	*BMP6*	*LOC694405*	*NIPSNAP3B*	*PXK*	*LPIN1*	
DNASE1	*SIGLEC9*	*EFEMP1*	*CYB5R3*	*CCL26*	*ANK3*	
KCNB2	*MAB21L1*	*IGHV4OR15-8*	*VASH2*	*PBX3*	*SESN3*	
SSBP1	*RTFDC1*	*OLR1*	*S100A10*	*C15H9orf3*	*CCDC126*	
KCNJ13	*PPARGC1A*	*SERTAD4*	*OAS1*	*APBB2*	*cDNA IMAGE:3565734*	
