# Supplementary material for: Whole exome analysis of patients in Japan with hearing loss reveals high heterogeneity among responsible and novel candidate genes
Source: Orphanet J Rare Dis. 2022 Mar 5;17:114. doi: 10.1186/s13023-022-02262-4 (PMC8898489; doi:10.1186/s13023-022-02262-4)
Supplement: Supplementary file 4 — Additional file 4. List of captured regions with insufficient average read depths (<20) of Tier 1 genes in this study. [file 13023_2022_2262_MOESM4_ESM.pdf]

Additional file 4. List of captured regions with insufficient average read depths (<20) of Tier 1 genes in this study.

Genomic position	Gene Symbol	Exon	Coding region
chr1:21616854_21616909	*ECE1*	2	NM_001397.3:c.51_137
chr3:46742859_46743072	*TMIE*	1	NM_147196.3:c.1_93
chr11:17568918_17569093	*OTOG*	1	NM_001277269.2:c.1_174
chr12:133195400_133195577	*P2RX2*	1	NM_170682.4:c.1_173
chr19:6361583_6361785	*CLPP*	1	NM_006012.4:c.1_198
