# Supplementary material for: Whole exome analysis of patients in Japan with hearing loss reveals high heterogeneity among responsible and novel candidate genes
Source: Orphanet J Rare Dis. 2022 Mar 5;17:114. doi: 10.1186/s13023-022-02262-4 (PMC8898489; doi:10.1186/s13023-022-02262-4)
Supplement: Supplementary file 5 — Additional file 5. Primers used in this study. [file 13023_2022_2262_MOESM5_ESM.pdf]

Additional file 5. Primers used in this study.

Gene symbol	Reference mRNA	Targeted exon	Fwd primer (5'-3')	Rev primer (5'-3')
MYO15A	NM_016239.3	46	tgtaaaacgacggccagtTTGGCAATGCCTGACACACT	caggaaacagctatgaccACTTGGTAAGTGGCTGTGGAG
MYO15A	NM_016239.3	47	gtaaaacgacggccagtGGTAACGGAAAGCGTGAAGC	caggaaacagctatgacAGTAAGGTGTCACTGCTGGC
MYO15A	NM_016239.3	51	tgtaaaacgacggccagtCCGCCAGTTGGTGAAGTCTA	caggaaacagctatgaccGGGCAAACTCGAGCATTGTG
MYO15A	NM_016239.3	2	gtaaaacgacggccagtCAAACGGAGCCTGAAGGGG	caggaaacagctatgacAGTCGTAGGGATCCTCGGG
MYO15A	NM_016239.3	2	gtaaaacgacggccagtACGACTACTACCACCCCGAC	caggaaacagctatgacGTCCTCTTCTTCGTCCGCAT
MYO15A	NM_016239.3	59	gtaaaacgacggccagtTGAATGGGAGACACAAGCCC	caggaaacagctatgacAAAGGCGCACAATCCTCTGA
MYO15A	NM_016239.3	61	gtaaaacgacggccagtTTGGAGTTATTACCATCTGTGGTC	caggaaacagctatgacAGAAGCCTACGTGTGCTGAC
CDH23	NM_022124.5	8	tgtaaaacgacggccagtAGCCCTTCCCTGCTGGAGTG	caggaaacagctatgaccGGAGATGGGCGTGGTGTGAA
CDH23	NM_022124.5	55	tgtaaaacgacggccagtGTGGCCTTCCTTGGACACCC	caggaaacagctatgaccGCACAGCCCATATAGTAACCACTGTTC
CDH23	NM_022124.5	9	tgtaaaacgacggccagtATGCCTGAGCCCTTGGCATC	caggaaacagctatgaccCCCAAAGTGCCCAACCGTCT
PDZD7	NM_001195263.1	4	gtaaaacgacggccagtGGCCAACCCTCAAGTCAGAA	caggaaacagctatgacAGTGGGTTTTGGGTGTTGGT
OTOF	NM_001287489.1	46	caggaaacagctatgaccAACCCTGGCTCCAGCATTCA	tgtaaaacgacggccagtGGGCAAGAGAGACCCATTCCA
OTOG	NM_001277269.1	35	tgtaaaacgacggccagtGCTATCCACAGAGAAGGGCG	caggaaacagctatgaccCTGCTGGAGTGAAAGGCAGT
OTOG	NM_001277269.1	17	tgtaaaacgacggccagtGCTATTTGTGCCCCCTGAGT	caggaaacagctatgaccTCTCTCCTTTCCCCTCCCTG
MYO6	NM_004999.3	16	tgtaaaacgacggccagtTGCTATGTTGTTTCTGATCAGTCC	caggaaacagctatgaccTCTAAGGAAGATACTGTGCTGGA
MYO6	NM_004999.3	13	tgtaaaacgacggccagtTGTTTAGGTGCACTCTGTGGC	caggaaacagctatgaccTGAGGTTGGCCTGTAAGTTAGG
PTPN11	NM_002834.3	13	tgtaaaacgacggccagtTGGCAAGTGAGGGAATCCTGA	caggaaacagctatgaccATCCAAGAGGCCTAGCAAGAGAA
PTPN11	NM_002834.3	7	tgtaaaacgacggccagtAAGAAGTAATGCTGATCCAGGC	caggaaacagctatgaccTGCTAACAAGAGCACACGACC
PTPN11	NM_002834.3	13	gtaaaacgacggccagtCCTGACTTCTGCCACTTCGT	caggaaacagctatgacCTTGCCACATGAGCCTGAGA
SOX10	NM_006941.3	3	tgtaaaacgacggccagtTCTCACCCTCCAGCCCATGA	caggaaacagctatgaccTGCCATCCAGCCATCTCCTG
SOX10	NM_006941.3	4	tgtaaaacgacggccagtCCCGACTCATGCTGCCAAA	caggaaacagctatgaccCCCGACCTGTCAGCCTCTTCA
EYA1	NM_000503.5	12	caggaaacagctatgaccGCAGCTGTTGCCCAAGTCTCTC	tgtaaaacgacggccagtTGCAAGTGTTACGAATTATGTTGGACA
ZNF335	NM_022095.3	9	gtaaaacgacggccagtACCGCAAGTATGTGGAGCAG	caggaaacagctatgaccACACGCGTTCTGAAGAGCA
BAIAP2L2	NM_025045.5	7	gtaaaacgacggccagtCAGTCAGGAAGAACAGGGGG	caggaaacagctatgacAGAGGGAAGAAGGGAGGGAA
HKDC1	NM_025130.3	4	tgtaaaacgacggccagtGTCCAAGTTCCGAGTGCTGA	caggaaacagctatgaccATTCTACTCCCCCGAGCTGT
HKDC1	NM_025130.3	12	tgtaaaacgacggccagtACGTCTCCTCTTCCTCCCAA	caggaaacagctatgaccGAAGGCAGGCTGGGAGTTAC
SVEP1	NM_153366.3	38	gtaaaacgacggccagtCCCGGGCTATAAGTCAGTCG	caggaaacagctatgacCCTCCAAGCCAGTGACCATT
SVEP1	NM_153366.3	44	gtaaaacgacggccagtTTCTTGGTGTCCTACAGACTGC	caggaaacagctatgacAACACTCCTCAGGAAACCCTC
CACNG1	NM_000727.3	4	tgtaaaacgacggccagtACATGGTTGCCCTTCTGAGG	caggaaacagctatgaccGAGCAGGGTGGGGCTAAAAT
GTPBP4	NM_012341.2	9	tgtaaaacgacggccagtAGATGTTCACATTTGCAGCCTCC	caggaaacagctatgaccAAGGGTCTGTCTTCACGCC
PCNX2	NM_014801.3	27	tgtaaaacgacggccagtGCTTAAAACTTCGCTGGCCC	caggaaacagctatgaccTGTGCATTACACCCAGCAGA
PCNX2	NM_014801.3	19	tgtaaaacgacggccagtGGATGCTCTTCTTAGGCCCC	caggaaacagctatgaccCATCCCTCTCCCCATCCTCT
TBC1D8	NM_001102426.1	12	gtaaaacgacggccagGGTGTGAGGTAGGGGCTAGT	caggaaacagctatgacTCCTCAGCATTGGCCTCAAG
qPCR primers for copy number analysis				
STRC	NM_153700.2	19	CCAGCTCCACCTGAATCCTG	CTCTGGAGCTCTTTGCACCA
STRC	NM_153700.2	5' upstream	AGGGAGAGAATGGGGAAGGG	CCCCAAAGGCCTTCACTTTAT
MYO7A	NM_000260.3	10	CAGCACGCACATTTGAAAACC	GACCTCAAGCAGGATGCAG

Nucleotides in lower case indicate sequencing primers.
