# Supplementary material for: Whole exome analysis of patients in Japan with hearing loss reveals high heterogeneity among responsible and novel candidate genes
Source: Orphanet J Rare Dis. 2022 Mar 5;17:114. doi: 10.1186/s13023-022-02262-4 (PMC8898489; doi:10.1186/s13023-022-02262-4)
Supplement: Supplementary file 9 — Additional file 9. Variants of novel candidate genes associated with hearing loss. [file 13023_2022_2262_MOESM9_ESM.pdf]

Additional file 9. Variants of novel candidate genes associated with hearing loss.

Family ID	Gene symbol	Tier	Reference mRNA	Nucleotide change	Amino acid change	Genomic position (GRCh37)	dbSNP ID	MAF (1000G)	MAF (ESP6500)	MAF (ExAC)	MAF (gnomAD)	MAF (HGVD)	MAF (in house)	REVEL	CADD	UCSC conservation	Splice site (MaxEntScan)	Splice site (HSF3.0)
1427	*BAIAP2L2*	2	NM_025045.5	c.506T>C	p.(Val169Ala)	chr22:38493145A>G	none	0	0	0	0	0	0	0.287	25.8	11/11, 42/48, 20/27	-	-
1676	*HKDC1*	3	NM_025130.3	c.376-2A>G	p.? (splice mut)	chr10:70992768A>G	none	0	0	0	0	0	0	-	33.0	-	11.49 to 3.54	Affected (-31.14%)
1676	*HKDC1*	3	NM_025130.3	c.1771A>C	p.(Lys591Gln)	chr10:71010343A>C	rs376814835	0	8.242E-06	0	0	0	0	0.708	23.2	12/12, 47/48, 36/37	-	-
1535	*SVEP1*	4	NM_153366.3	c.6766C>G	p.(Pro2256Ala)	chr9:113171114G>C	none	0	0	0	0	0	0	0.622	23.7	11/11, 50/50, 32/36	-	-
1535	*SVEP1*	4	NM_153366.3	c.7357G>A	p.(Val2453Met)	chr9:113170523C>T	rs374113606	0	2.000E-04	8.273E-05	4.819E-05	0.002	0.004	0.264	25.6	11/11, 50/50, 37/37	-	-
1555	*SVEP1*	4	NM_153366.3	c.6977C>T	p.(Pro2326Leu)	chr9:113170903G>A	rs369710771	0	8.300E-05	1.160E-04	9.274E-05	0.001	0.001	0.589	25.0	11/11, 50/50, 35/36	-	-
1555	*SVEP1*	4	NM_153366.3	c.10294T>C	p.(Tyr3432His)	chr9:113141741A>G	none	0	0	0	4.018E-06	4.132E-04	0	0.235	23.0	11/11, 50/50, 20/33	-	-
1669	*CACNG1*	4	NM_000727.3	c.461C>T	p.(Ser154Leu)	chr17:65052179C>T	rs866479653	0	0	0	0	0	0	0.683	26.3	12/12, 43/45, 37/37	-	-
1696	*GTPBP4*	4	NM_012341.2	c.967C>G	p.(Leu323Val)	chr10:1051812C>G	none	0	0	0	0	0	0	0.23	16.4	12/12, 48/48, 32/32	-	-
1685	*PCNX2*	4	NM_014801.3	c.3505C>T	p.(Arg1169Trp)	chr1:233296041G>A	rs150235720	0	0.002	0.001	1.38E-03	0	0	0.139	25.5	12/12, 45/47, 20/36	-	-
1685	*PCNX2*	4	NM_014801.3	c.4777C>T	p.(Arg1593Ter)	chr1:233152729G>A	rs751554663	0	0	1.654E-05	1.204E-05	0	0	-	42.0	-	-	-
1575	*TBC1D8*	4	NM_001102426.1	c.1997C>T	p.(Ser666Leu)	chr2:101646133G>A	none	0	0	4.130E-05	4.012E-05	0	0	0.569	29.4	12/12, 49/49, 36/38	-	
